# Extrusion 3D Printing of Polymeric Materials with Advanced Properties

**DOI:** 10.1002/advs.202001379

**Published:** 2020-08-05

**Authors:** Zhen Jiang, Broden Diggle, Ming Li Tan, Jekaterina Viktorova, Christopher W Bennett, Luke A. Connal

**Affiliations:** ^1^ Research School of Chemistry Australian National University Canberra ACT 2601 Australia

**Keywords:** 3D printing, 4D printing, additive manufacturing, polymers

## Abstract

3D printing is a rapidly growing technology that has an enormous potential to impact a wide range of industries such as engineering, art, education, medicine, and aerospace. The flexibility in design provided by this technique offers many opportunities for manufacturing sophisticated 3D devices. The most widely utilized method is an extrusion‐based solid‐freeform fabrication approach, which is an extremely attractive additive manufacturing technology in both academic and industrial research communities. This method is versatile, with the ability to print a range of dimensions, multimaterial, and multifunctional 3D structures. It is also a very affordable technique in prototyping. However, the lack of variety in printable polymers with advanced material properties becomes the main bottleneck in further development of this technology. Herein, a comprehensive review is provided, focusing on material design strategies to achieve or enhance the 3D printability of a range of polymers including thermoplastics, thermosets, hydrogels, and other polymers by extrusion techniques. Moreover, diverse advanced properties exhibited by such printed polymers, such as mechanical strength, conductance, self‐healing, as well as other integrated properties are highlighted. Lastly, the stimuli responsiveness of the 3D printed polymeric materials including shape morphing, degradability, and color changing is also discussed.

## Graphical abstract

3D printing is a rapidly growing technology that has an enormous potential to impact a wide range of industries. This review focuses on material design strategies to achieve or enhance the 3D printability of functional materials. Specific emphasis is on extrusion based 3D printing techniques and the functional devices that can be prepared.

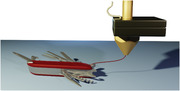

## 1 Introduction

Additive manufacturing (AM), the formal term for 3D printing, was first introduced in the 1980s by Hull.^[^ ^1^ ^]^ Since then, fabrication methods have been revolutionized, which has further intensified the influence of polymers on our society. This technology has become the leading rapid prototyping technology for fabricating highly defined complex 3D constructs.^[^ ^2^, ^3^, ^4^, ^5^, ^6^, ^7^, ^8^ ^]^ Additionally, a broad range of sizes from micrometer to meter scale can be produced. The price of commercially available 3D printers has reduced significantly (less than $500) leading to 3D printing of customized products at home, such as bicycle parts, jewelry, and electrical components. This technology has numerous benefits such as operational simplicity, customizable design, reliability, cost‐effectiveness, and diversity of the compatible materials, making it very promising for a wide range of technological applications such as biotechnology,^[^ ^9^, ^10^, ^11^, ^12^, ^13^ ^]^ energy/environment,^[^ ^14^, ^15^ ^]^ robotics,^[^ ^16^, ^17^ ^]^ and aerospace. Furthermore, when compared to other standard processing technologies, AM technology has the ability to fabricate a wide range of complex bioinspired structures with freedom in 3D shape design,^[^ ^5^, ^6^ ^]^ such as multilayered tubular structures,^[^ ^18^ ^]^ microfibril structures,^[^ ^19^ ^]^ cellular structures,^[^ ^20^, ^21^ ^]^ as well as various internal and external anatomical architectures.^[^ ^22^ ^]^

3D printing, is the conversion of a computer‐generated model into a real, physical object. The model is first converted into a series of triangulated coordinates which can be read by a printer and then transformed to a series of sequential layers. A computer‐controlled translation stage then moves based on the designed pattern, either in the form of laser optics or an ink based printhead, to fabricate objects one layer at a time. Various 3D printing technologies used in fabricating advanced polymers have been well reviewed and summarized,^[^ ^23^ ^]^ namely light‐based 3D printing^[^ ^24^, ^25^, ^26^ ^]^ extrusion‐based printing,^[^ ^27^, ^28^ ^]^ inkjet 3D printing,^[^ ^29^ ^]^ and powder bed fusion‐based methods.^[^ ^30^ ^]^ Extrusion‐based printing is widely used due to its simple printing mechanism and low‐cost of fabrication. The first step of this approach is the extrusion of materials through a nozzle with the aid of a mechanical force. The extruded and deposited polymer then undergoes solidification through mechanisms such as crystallization, chain rearrangement, recovery of noncovalent bond, or chemical crosslinking. After the completion of a single layer, the extrusion head either moves up or the build platform moves down for the deposition of the next layer. Extrusion‐based printing has been commercialized by several companies,^[^ ^23^ ^]^ hence a wide selection of cheap 3D printers are available for researchers. This technology has also been adopted into various fields and has been used in several areas such as energy devices,^[^ ^15^ ^]^ biomaterials,^[^ ^28^ ^]^ and soft actuators.^[^ ^16^, ^31^ ^]^

The printable materials or “inks” are very crucial components that are specific to the printing method and determine the performance of the final 3D‐printed products. Due to their processability and low cost, polymeric materials are by far the most utilized class of materials for 3D printing.^[^ ^23^ ^]^ The improvement of 3D printable polymeric materials is driving growth in this field. Many types of polymers including thermoplastics, thermosets, elastomers, hydrogels, functional polymers, polymer blends, and composites can be processed by 3D extrusion printing.^[^ ^23^, ^32^ ^]^ However, it is still a challenge to print polymers with advanced properties such as stimulus‐responsiveness, self‐healing, high mechanical strength, or other integrated properties. This is due to the lack of efficient and general approaches to tune rheological parameters of the polymer. Theoretically, most polymer solutions exhibit non‐Newtonian shear‐thinning behavior, which is one of the important properties considered for 3D extrusion printing. However, tough polymeric materials consisting of a stiff backbone chain or side group would show extremely high yield stress, hence very high applied pressures are required to induce flow through a nozzle. The 3D printing of self‐healable polymers containing dynamic covalent bonds^[^ ^33^ ^]^ also face a similar dilemma. In self‐healable systems, polymer chains would become entangled and viscosity increased after removing applied stress. The relaxation process is usually too lengthy (taking several minutes to hours) to be considered for real applications. The use of noncovalent bonds presents an attractive strategy to construct polymeric materials with a number of unique properties such as shape memory,^[^ ^34^ ^]^ stimulus‐responsiveness,^[^ ^35^, ^36^, ^37^, ^38^ ^]^ good processability,^[^ ^39^, ^40^ ^]^ toughness,^[^ ^41^ ^]^ and self‐healing.^[^ ^42^, ^43^ ^]^ However, due to its highly dynamic nature and relatively weak strength, the extruded filaments could easily undergo fast dynamic exchange, leading to short‐term stability as well as relatively poor mechanical properties of printed objects. Furthermore, material properties such as mechanical performance can be altered or compromised after formation into 3D structures, due to anisotropic effects caused by the directionality of the printed layers.

In this review, we focus on summarizing the state‐of‐the‐art advances in 3D extrusion‐based printing from a material‐focused standpoint as well as highlighting the new material properties of these novel printed devices. This review is divided into two parts: 1) Material design strategies to achieve or enhance the 3D printability of various polymeric systems by extrusion‐based methods and 2) Advanced material properties exhibited by the printed polymers. We focus on discussing how 3D printing technology could be utilized to enhance material properties and add function to the 3D printed objects. Throughout this review, the current challenges, perspectives, and future work required for achieving novel properties of 3D printable polymers are discussed.

## 2 Material Designs for 3D Printability

**Figure 1:**
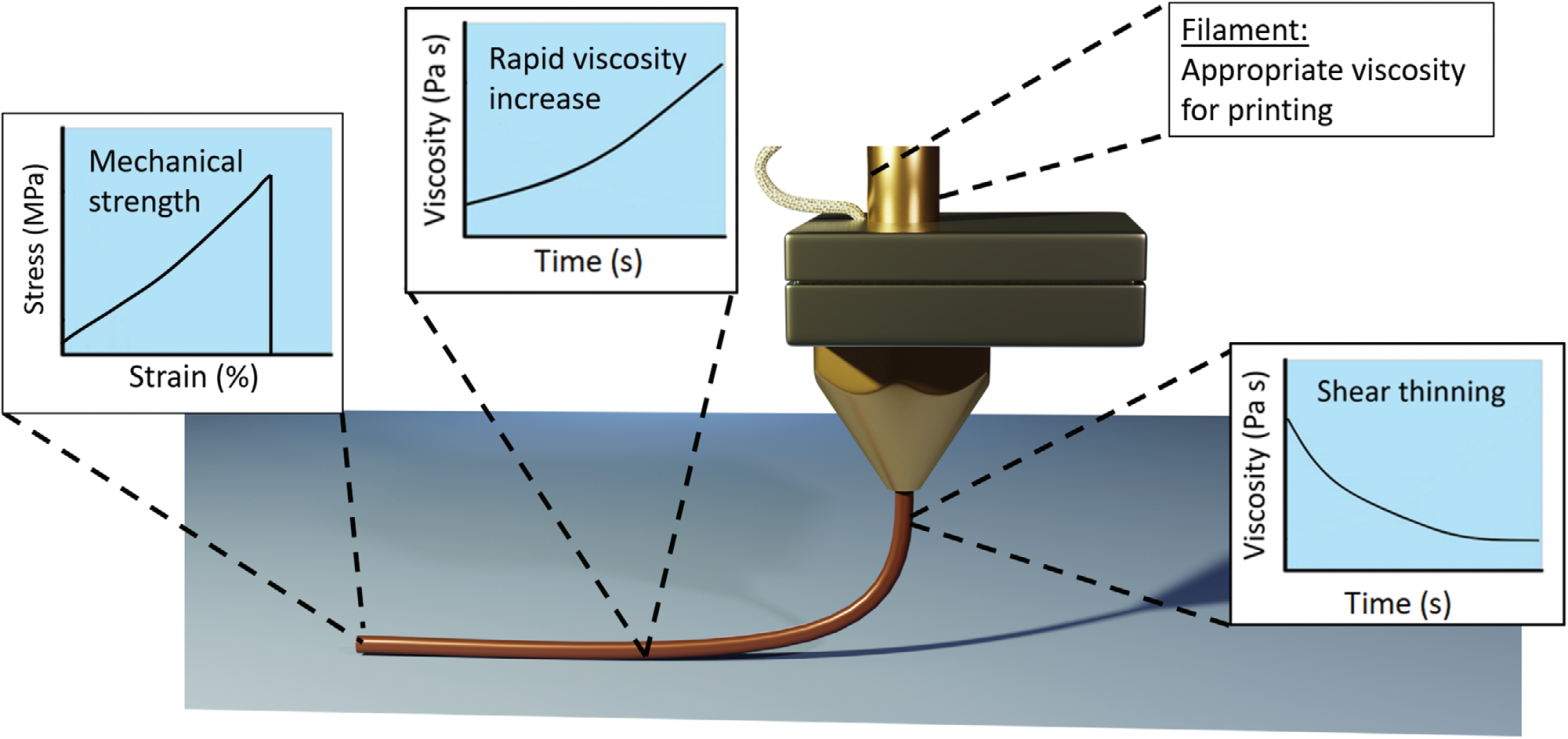
Key properties required for 3D extrusion printing of polymers. For DIW printable polymers, the appropriate viscosity for the polymer to be extrudable and be able to form continuous filaments is required. Once extruded, it needs to thicken to provide sufficient mechanical strength to support the printed structures and prevent delamination.

3D extrusion printing encompasses both fused filament fabrication (FFF) and direct ink writing (DIW).^[^ ^23^ ^]^ FFF is typically used for 3D extrusion of thermoplastic polymers, which are mechanically extruded as thin filaments through a heated nozzle into the extrusion print head.^[^ ^44^, ^45^ ^]^ To facilitate flow and deposition, the thermoplastic material is heated above the melting temperature for semicrystalline polymers or above the glass transition temperature for amorphous polymers. Once the filament has exited the nozzle, it cools down and the polymer's viscosity increases sharply, which retains the shape. DIW is used to form 3D materials from viscoelastic polymers. This process has been used to manufacture a variety of objects with a wide range of sophisticated geometries, sizes, and materials.^[^ ^27^ ^]^ During DIW process, the viscoelastic ink is dispensed out of a small nozzle a few hundred micrometers in diameter by external pressure and then deposited along digitally defined paths to fabricate 3D structures. Extrusion efficiency and 3D structure quality are highly dependent on the viscoelastic properties of polymeric inks, hence careful design of material properties and printing parameters are required. The extruded inks would also undergo chain reorganization and relaxation, so further physical or chemical treatment is typically needed to enhance its structural integrity. Rheological properties such as viscosity, yield stress, and elastic modulus need to be measured to determine the printability window of inks. For polymers to be printable via the extrusion method, the following properties are required (**Figure** **1**): 1) the polymers should be highly thixotropic and exhibit shear thinning behavior, 2) appropriate viscosity such that the material can be extrudable and form continuous filaments to ensure structure fidelity, 3) undergoes a rapid viscosity increase after extrusion to retain the 3D printed shape, 4) sufficient mechanical strength after exiting the nozzle is required to support the subsequently printed structures and to prevent delamination during and after printing.

### 2.1 Enhancing 3D Printability by FFF

In the following sections, we summarize material design strategies to overcome the printability challenges using the FFF technique with several classes of polymers, including thermoplastics, thermosets, and other polymeric materials.

#### 2.1.1 Thermoplastic Polymers

A thermoplastic polymer is a class of material governed by its thermal properties, which often consists of reversible and weak van der Waals forces. The material softens when heated above its glass transition temperature and behaves like a fluid. This process is completely reversible as no chemical bonding takes place during this process. This interesting property enables the polymers to be re‐molded and recycled into any desired shape by various processing technologies, such as injection molding, compression molding, and calendering. As such, thermoplastic polymers are very suitable for FFF. The significantly reduced viscosity upon heating facilitates extrusion through a nozzle. Once extruded, the filament cools and the viscosity increases sharply. The polymer rapidly solidifies and holds its shape.

Using 3D extrusion techniques, several commercial products have been formed from filaments. However, the extruded filaments of some polymers could easily undergo shrinkage. Thus, high fidelity of the fabricated 3D printed structures could be compromised. Some of these polymers are also semicrystalline and exhibit a low melt viscosity that can hamper a solid's dispersion in the filaments prepared by melt extrusion. Therefore, the design and development of new thermoplastic materials with enhanced 3D printability has emerged as an attractive and efficient way to overcome this limitation and extend its application range.

Historically one of the most utilized polymers for FFF 3D printing has been acrylonitrile−butadiene−styrene (ABS) copolymers.^[^ ^46^ ^]^ ABS exhibits several interesting features such as good mechanical properties, high fluidity, low glass transition temperature, and a wide processing window. These key properties make it an excellent material for 3D extrusion printing. Whilst ABS polymers have been used in commercial 3D printer products, pure ABS was found to show large shrinkage, compromising structural fidelity. Incorporating reinforced nanomaterials such as carbon fibers,^[^ ^47^ ^]^ glass fibers,^[^ ^48^ ^]^ graphene,^[^ ^49^ ^]^ or carbon nanotubes^[^ ^50^ ^]^ into thermoplastic materials to form composites has been demonstrated as the most widely used method for improving dimensional stability, due to enhanced toughness that these materials contribute when added. Zhong et al. demonstrated that the amount of shrinkage was decreased and the surface rigidity was improved when short glass fibers reinforced the ABS composite, resulting in a much improved 3D printability.^[^ ^48^ ^]^ However, the flexibility and handleability of the 3D printed composite objects were found to be compromised. This was then addressed by adding a small amount of plasticizer and compatibilizer. Similar observations were also made when ABS was composited with carbon fiber^[^ ^47^ ^]^ and carbon nanotubes,^[^ ^50^ ^]^ in which the increase of tensile strength and Young's modulus were at the expense of toughness, yield strength, ductility and breaking elongation. Sun and co‐workers^[^ ^49^ ^]^ found that the incorporation of graphene strongly affected the printability. Too much graphene in the system resulted in discontinuous extrusion and material inhomogeneity due to polymer aggregation.

The addition of reinforced nanomaterials and small molecular plasticizers have also been demonstrated to enhance the 3D printability of other important and widely used thermoplastic materials such as polyurethane (TPU)^[^ ^51^ ^]^ and poly(lactic acid) (PLA).^[^ ^52^ ^]^ Wang and co‐workers demonstrated FFF printing of poly(vinyl alcohol) (PVA), which is an important class of water‐soluble polymers with many interesting properties including low toxicity, hydrophilicity, as well as excellent mechanical properties. PVA is traditionally very difficult to print due to its large number of hydrogen bonds. In order to process PVA, glycerol, water,^[^ ^53^ ^]^ and ionic liquids,^[^ ^54^ ^]^ were introduced to reduce the melting point. It was suggested that the compressive modulus and apparent viscosity are important parameters to maintain good printability. Furthermore, incorporating nanofillers or other polymers not only enhances the structural fidelity but also offer additional multifunctionalities such as biocompatibility^[^ ^55^ ^]^ and conductivity^[^ ^51^, ^52^ ^]^ into 3D printed devices.

**Figure 2:**
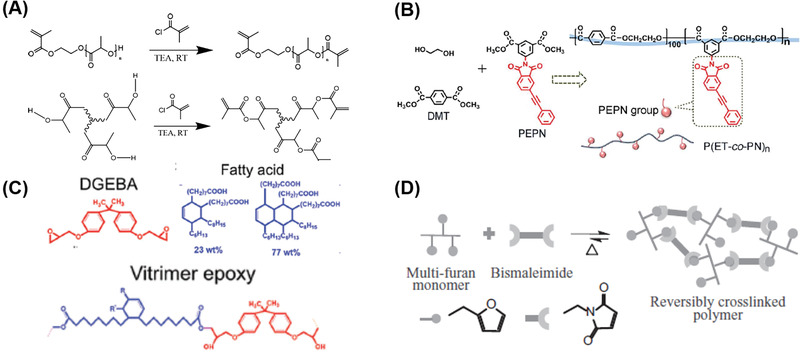
Examples of the material design strategies for printing A,B) thermoplastic and C,D) thermosets via FFF: A) Synthetic route of incorporating dynamic *π*–*π* stacking networks into poly(ethylene terephthalate). Reproduced with permission.^[^ ^59^ ^]^ Copyright 2019, Royal Society of Chemistry. B) The design and synthesis of low‐molecular‐weight surface‐segregating additives for promoting adhesions between printed layers. Reproduced with permission.^[^ ^58^ ^]^ Copyright 2019, American Chemical Society. C) Chemical structures of the major components of ink formulations, in which the epoxy group could undergo the thermal curing to enhance the ink stability during printing process. Reproduced with permission.^[^ ^60^ ^]^ Copyright 2019, Royal Society of Chemistry. D) Furan‐maleimide Diels–Alder chemistry was used to create 3D printable thermoset polymers. Reproduced with permission.^[^ ^61^ ^]^ Copyright 2017, John Wiley and Sons.

Anisotropy and poor interlayer adhesion are the major challenges limiting the further practical applications of FFF technology. Levenhagen and Dadmun addressed these issues by using low‐molecular‐weight surface‐segregating additives (LMW‐SuSAs).^[^ ^56^, ^57^ ^]^ These LMW‐SuSAs preferentially segregates to the interface, leading to increased entanglement between adjacent printed layers. The bonding and adhesion of printed layers could be tunable through controlling the molecular weight and architecture of the additive. Such improved diffusion and entanglement of adjacent layers lead to nearly identical moduli regardless of print orientation. The same group also recently reported the incorporation of UV‐curable methacrylate groups into LMW‐SuSAs to initiate crosslinks via light irradiation between printed layers (**Figure** **2**A).^[^ ^58^ ^]^

Polymer blending strategies could also improve the 3D printability. Recently, Feng and co‐workers incorporated poly(methyl methacrylate) (PMMA) and methacrylate–butadiene–styrene (MBS) into ABS.^[^ ^62^ ^]^ The melt mass‐flow rate (MFR) was measured to determine the material's suitability for extrusion. If the MFR value is low, the nozzle will be blocked, interrupting the printing process. On the contrary, a high MFR value signifies lack of support of the extruded material, which harms dimensional accuracy. A higher MFR value was observed in an ABS/PMMA blend, indicating that PMMA can increase the MFR, thus improving the 3D printability of ABS. Even though incorporating MBS into the system slightly reduced the MFR the material was still extrudable. Styrene–ethylene–butadiene–styrene (SEBS) and ultrahigh molecular weight polyethylene (UHMWPE) were also blended with ABS,^[^ ^63^ ^]^ which was demonstrated to provide high toughness, low melt viscosity, and low processing temperature in 3D printed products. Poly(butylene succinate) (PBS) could not form effective continuous monofilaments suitable for extrusion. However, through blending with PLA, the melt strength of PBS increases from 67 to 564 MPa with the increasing content of PLA from 0 to 60 wt%.^[^ ^64^ ^]^ This 3D printed blend possessed higher melt viscosity, larger tensile strength, and Young's modulus. The distortion or detachment due to residual stress caused by volume shrinkage during the printing process was resolved with blending, resulting in good dimensional accuracy and pearl‐like gloss. 3D extrusion printing of poly(2‐vinylpyridine) (P2VP) which is a widely studied pH sensitive polymer, has been demonstrated.^[^ ^65^ ^]^ Fragmentation was observed when these fragile filaments of pure P2VP were pushed through the nozzle. The printability was improved after blending with 12 wt% thermoplastic ABS, which was demonstrated by a well‐defined star‐fish and seahorse structures extruded at temperature ranging from range 145−195 °C. Thermal processing parameters were also optimized in order to achieve high quality 3D‐printable filaments.

Additive‐free 3D printing of thermoplastic polyesters was also achieved by controlling the side chain structures. Most recently, Joy and co‐workers systematically studied the relationship between saturated, aliphatic pendant side chain length and 3D printability.^[^ ^66^ ^]^ It was found that an increase of side chain length could reduce viscosity and enable extrusion at low temperature and pressure, while long chain length could adversely affect the ability to retain a 3D printed shape. In another work, the commonly overlooked issue of possible degradation in thermoplastic aliphatic polyesters during FFF process was studied. Jain et al. found that biodegradable polymer's structure and properties could significantly affect 3D printability.^[^ ^67^ ^]^ It was demonstrated that degradation of poly(l‐lactide) (PLLA) could be mitigated by increasing molecular weight.

Chemical modification of thermoplastic polymers with supramolecular interactions is another approach to enhance 3D printability. For example, 3D printing of poly(ethylene terephthalate) (PET) is challenging because of rapid crystallization after extrusion and subsequent weak adhesion between layers. Recently, Wang and co‐workers reported that the incorporation of phenylacetylene (PEPN) groups featuring *π*–*π* interactions at side chain (Figure 2B).^[^ ^59^ ^]^ Recrystallization upon cooling down is prevented due to the destroyed chain regularity by pendant PEPN. The decreased differential temperature between melting temperature (*T*_m_) and glass transition temperature (*T*_g_) also enabled rapid solidification during the 3D printing process. The use of hydrogen bonding has been reported to improve the 3D printability of a poly(isobutylene) (PIB) system.^[^ ^68^ ^]^ The viscosity of the polymer could be can be widely tunable by temperature due to the breaking and reforming of the hydrogen bonds. It was found that molecular architecture could affect 3D printability. For example, the three‐armed star polymer has poor 3D printability due to its hydrogen‐bonded network.

#### 2.1.2 Thermoset Polymers

Thermoset polymers are very important materials that have been utilized in various high‐technology applications, due to the stable thermomechanical properties in the form of excellent chemical and thermal resistance. In contrast to thermoplastic polymers, thermoset polymers cannot be re‐melted for reshaping after thermal curing because of the formation of irreversible chemical crosslinking. Most of the chemically crosslinked thermoset materials are mechanically tough and have a high (*T*_g_) enabling them to withstand high processing temperatures. They are typically unable to flow under applied pressure, making it extremely challenging to use extrusion methods to fabricate 3D thermoset objects. Jerry Qi and co‐workers demonstrated that composite vitrimer‐containing epoxy inks formulated with a rheological modifier such as a nanoclay, could easily be extruded from the nozzle and retain sufficiently high viscosity after extrusion. However, 3D printed parts can still undergo deformation or collapse under a high curing temperature, compromising structural fidelity. To tackle this challenge, further chemical modification was required, so the viscosity of the system before extrusion (Figure 2C) was increased to avoid shape collapse at the curing temperature.^[^ ^60^ ^]^ The authors further treated the printed objects with a two‐step curing process (pre‐cured at 60 °C for 20 h, and then fully cured for 6 h at 130 °C under vacuum). The excellent printability of this modified epoxy system was demonstrated with its ability to produce diverse and complex structures. Chandrasekaran et al. also reported the use of a cyclotrimerization reaction to enhance the thermal stability of rheologically tailored cyanate ester (CE) resin ink.^[^ ^69^ ^]^ Thermo‐oxidative stability shows no obvious change in mass of the fully cured, printed CE resins at temperatures up to 400 °C. Incorporating a salt as a sacrificial carrier material into the precursor inks represent another efficient approach to enhance the 3D printability of thermoset polymers.^[^ ^70^ ^]^ The 3D‐printed shape before and during curing could be well retained as the sacrificial carrier materials can serve as aggregates to provide mechanical support.

Incorporating dynamic covalent chemistry is an attractive approach to improve the processing properties of thermoset polymers by FFF.^[^ ^71^ ^]^ Dynamic covalent chemistry provides a promising solution to overcome the barrier for reprocessing or reshaping products, an on‐going problem for thermoset polymer processing. The use of dynamic chemistry enables reversible crosslinking, which has the potential to improve the 3D printability of thermoset polymers. Voit and co‐workers developed new polymers that crosslinked with furan‐maleimide through Diels–Alder chemistry and studied the 3D printability as well as printing quality of the resulting 3D structures.^[^ ^61^ ^]^ Due to the thermally reversible crosslinking between the furan and bismaleimide groups (Figure 2D), the viscosity of the system could be tuned by changing the temperature. The polymers were extruded at high temperatures with the viscosity as low as 0.8 Pa s. To increase the viscosity the polymer was subsequently treated by a constant flow of cool air.

In short, FFF allows 3D printing of thermoplastic and thermoset polymers. For thermoplastics, printability was enhanced with additives such as nanomaterials, plasticizers or by chemical design. High temperature is needed for FFF 3D printing, hence potentially toxic vapor is generated when melting these polymers. Next generation FFF materials should minimize or eliminate toxic vapor release for this technology to be applicable in areas such as aerospace. Alternatively, DIW (discussed in the next section) could potentially be used for 3D printing in applications where emissions are a concern.

### 2.2 Enhancing 3D Printability by DIW

In the following sections, we summarize material design strategies to overcome the printability challenges using the DIW technique of several classes of polymers, including thermosets, hydrogels, and other polymeric materials.

#### 2.2.1 Thermoset Polymers

Lewis and co‐workers reported the first example of DIW 3D printing epoxy composite ink reinforced by chopped carbon fibers and inorganic nanomaterials (**Table** **1**).^[^ ^20^ ^]^ The pure resin was observed to lack shear‐thinning behavior, indicating poor 3D printability as the ink would immediately spread upon exiting the nozzle. A shear‐thinning effect was introduced after modification with nanoclay platelets, which enables a pronounced decrease in viscosity at a high shearing rate. The immediate viscosity increase with a low shearing rate was achieved, resulting in the stabilization of 3D printed shapes due to the recovery of the high viscosity after deposition. Furthermore, adding SiC whiskers could enhance the stiffness and toughness without compromising shear thinning behavior, which is very important for printing complex 3D structures. It was further demonstrated that a high aspect ratio (roughly 200 µm and 2 mm in height) for square, hexagonal, and triangular honeycomb structures were successfully fabricated in high fidelity. Using the same strategy, both high surface area colloidal silica and dispersed high aspect ratio, discrete carbon fibers have been added into the bisphenol‐F epoxy resin oligomer (BPFE) system.^[^ ^72^ ^]^ The BPFE was modified into a thixotropic, non‐Newtonian fluid followed by chemical crosslinking to stabilize 3D printed composite architectures. To retain its shape and enhance the temperature stability of the product following extrusion, Jerry Qi and co‐workers performed photopolymerization after each layer was applied, which avoids nozzle clogging and shape deformation problems.^[^ ^60^ ^]^ Complex 3D structures, without sagging or defects, were achieved with the formation of a stable network catalyzed by UV irradiation before thermal curing.

**Table 1:** Summary of material design approaches to enhance the 3D printability of thermosets; listed are the 3D printing method, tensile strength, and printing resolution

Polymer	Design approach	Printing methods	Tensile strength	Printing resolution	Post‐printing treatments	Ref.
Epoxy resins	Adding nanoclay platelets	DIW	69.8 ± 2.9 MPa	Roughly 200 µm and 2 mm in height	Cured at 100 °C for 15 h, followed by curing for 2 h at 220 °C	^[^ ^20^ ^]^
Bisphenol‐F epoxy resin oligomer	Adding carbon fibers	DIW	85 MPa	250 µm	Cured at 80 °C for 12 h followed by curing at 120 °C for 24 h	^[^ ^72^ ^]^
Polyester, epoxy, and polyurethane	Adding sodium chloride particles	FFF	150–470 kPa	micrometer‐sized porous structures	Cured at 100 °C for 12 h, followed by curing at 150 °C for 24 h	^[^ ^70^ ^]^
Vitrimer‐containing epoxy	Slight pre‐crosslinking precursor	FFF	4–12 MPa	Hundreds of micrometers	Cured at 60 °C for 20 h followed by curing at 130 °C for 6 h	^[^ ^60^ ^]^
Polymers containing furan‐maleimide linkages	Diels–Alder chemistry	FFF	Up to 26 MPa	561–1015 µm	6–8 °C for 8–12 h	^[^ ^61^ ^]^
Bisphenol type epoxy	Hydrogen bonding	DIW	–	200 mm	UV curing	^[^ ^64^ ^]^
Poly(dicyclopentadiene)	Frontal polymerization	DIW	50–55 MPa	–	Further solidified by frontal polymerization	^[^ ^73^ ^]^

Dynamic noncovalent hydrogen bonding could also be utilized to modify thermoset polymers for 3D extrusion printing. Nam and co‐workers added amides into a UV‐curable acrylated epoxy, which resulted in the formation of hydrogen bonds.^[^ ^74^ ^]^ The liquid acrylated epoxy was frozen after the addition of the donor octadecanamide. The newly formed hydrogen bonds could be broken under a shearing force, producing a low viscosity liquid. After extrusion, the observed immediate phase change to the solid state was due to rapid association of hydrogen bonding. This enables diverse layer‐by‐layer 3D structures to be easily printed without collapsing. The authors demonstrate a relatively low curing temperature and rapid curing speed, which very important for the formation of complex structures.

Conventional curing of thermosets has an energy cost, heating the monomers to high temperature for several hours. Sottos and co‐workers utilized frontal polymerization in which the heat energy could be generated from self‐propagating exothermic reaction for further propagation to cure dicyclopentadiene (DCPD).^[^ ^73^ ^]^Frontal polymerization technology also enables transformation of monomer solutions into fully cured polymers within seconds. The use of alkyl phosphite as inhibitors could substantially extend the room‐temperature liquid‐processing window up to 30 h, and also allows the tuning of rheological properties of formed viscoelastic gels over a wide range. Such gels exhibited shear thinning behavior which facilitates the extrusion from print head. Further curing was conducted upon exiting the nozzle resulting in the formation of stable free‐form complex architectures.

#### 2.2.2 Hydrogels

Hydrogels are an appealing class of polymeric 3D networks that are typically formed by hydrophilic chains swollen in an aqueous environment. They possess a wide range of material properties and functionalities including: high water‐content, shape changing, multistimuli responsiveness and biocompatibility.^[^ ^75^, ^76^, ^77^ ^]^ These materials are particularly attractive for numerous applications ranging from soft actuators^[^ ^78^ ^]^ and sensors to biological^[^ ^77^ ^]^ and energy applications.^[^ ^79^ ^]^ With the rapid development of additive manufacturing, hydrogels represent one of the most researched polymeric materials for 3D printing applications.^[^ ^80^ ^]^

**Figure 3:**
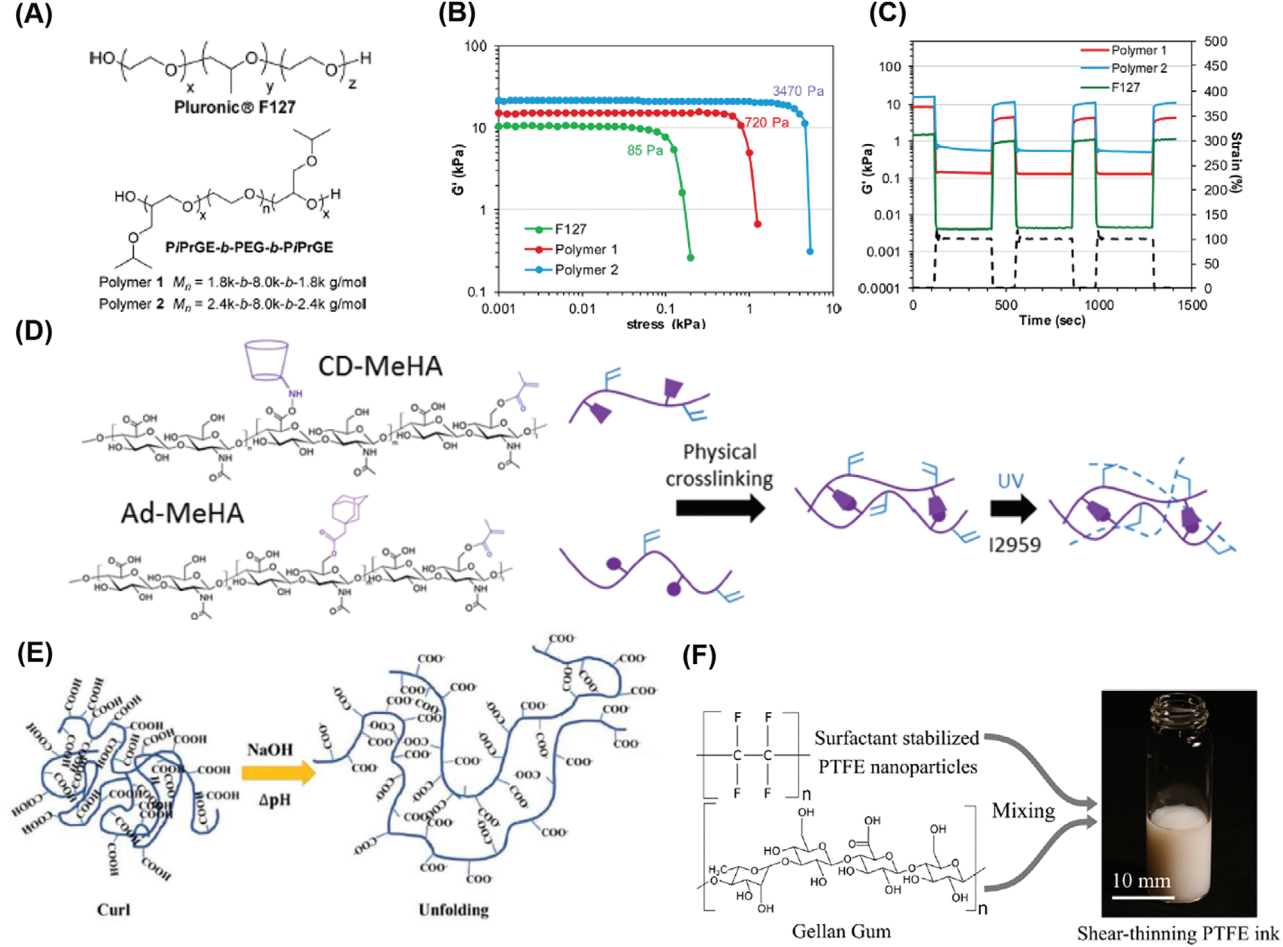
Examples of material design approaches toward A–E) printable hydrogels and F) PFTE via DIW. A–C) Hydrophobically crosslinked hydrogel. A) Chemical representations for PiPrGE‐*b*‐PEG‐*b*‐PiPrGE triblock copolymers and F127. Reproduced with permission.^[^ ^82^ ^]^ Copyright 2015, American Chemical Society. B) Storage modulus versus stress showing the corresponding yield stress for different polymer ink formulations. C) Cyclic shear‐thinning experiment of the polymers in (A). D) An example of double crosslinked hydrogels for 3D printing for improved resolution and stability: Hyaluronic acid modified with both methacrylates, guest and host molecules. Ad–MeHA and CD–MeHA macromers crosslink by both physical bonding upon mixing and through a secondary crosslinking of methacrylates with UV light exposure.^[^ ^86^ ^]^ Copyright 2015, John Wiley and Sons. E) Schematic of the fluid‐to‐gel transition of carbomer enabled ink through increasing molecular interactions upon pH change. Reproduced with permission.^[^ ^87^ ^]^ Copyright 2019, John Wiley and Sons. F) Schematics of the molecular structures and processes developed to 3D‐print PTFE structures. Reproduced with permission.^[^ ^88^ ^]^ Copyright 2019, American Chemical Society.

Hydrogels formed through noncovalent interactions between their building blocks for network formation are potentially suitable for DIW due to the dynamic nature of these physical associations. Shear‐thinning behavior has been demonstrated in such physical hydrogel systems because the weak physical bonding can be easily broken under applied pressure.^[^ ^81^ ^]^ Hydrogels can also have an immediate recovery to the solid state when the shear force is removed due to the highly mobile and transient noncovalent network. Nelson and co‐workers developed a novel poly(isopropyl glycidyl ether)‐*b*‐poly(ethylene glycol)‐*b*‐poly(isopropyl glycidyl ether) (PiPrGE‐*b*‐PEG‐*b*‐PiPrGE) hydrogel for DIW (**Figure** **3**A).^[^ ^82^ ^]^ The sol–gel transition is reversibly driven by thermo‐induced hydrophilic–hydrophobic changes of the PiPrGE block. Due to the decreased lower critical solution temperature (LCST) of the polymer solution, the physically crosslinked hydrogels were formed at room temperature through hydrophobic interactions stemming from the packing of the micelles. Rheological experiments suggest that the hydrogel viscosity significantly decreased as the shear rate increased, indicating the presence of non‐Newtonian fluids and shear‐thinning (Figure 3B). From the cyclic shear‐thinning experiments (Figure 3C) their hydrogels exhibited a marked decrease in the storage moduli at high strains and immediate recovery at low strains for each cyclic testing. These results suggest that such physical hydrogels are very suitable for DIW applications, which were indeed demonstrated as capable of printing free‐standing patterned structures with good fidelity. The authors suggested that the gel yield stress is also an important parameter to be considered for gel extrusion,^[^ ^83^ ^]^ as it provides an indirect indication of the gel's ability to support subsequent stacked layers during 3D printing. Nelson and co‐workers have also demonstrated that both dynamic and the static yield stress could be tuned by varying hydrogel formulations and are critical rheological parameters for printability.^[^ ^84^ ^]^ It was determined that materials with low dynamic yield stress below 200 Pa are not suitable for DIW, as they do not retain their shape well enough to form a filament upon extrusion. Gels of high dynamic yield stress afforded well extruded, continuous filaments. The physical hydrogels formed below upper critical solution temperature were also reported to be well suited for DIW printing.^[^ ^85^ ^]^ Similar to their counterpart hydrogels formed above LCST, the excellent shear thinning, rapid structure recovery and yield stress were demonstrated by comprehensive rheological measurements. Interestingly, such hydrogels could be used to enhance the 3D printability of alginate indicated by the increment of storage moduli up to 11 kPa.

Other types of hydrogels formed through noncovalent crosslinking, such as by ionic coordination,^[^ ^89^ ^]^ electrostatic interactions,^[^ ^90^, ^91^, ^92^, ^93^ ^]^ host–guest interactions,^[^ ^86^, ^94^, ^95^, ^96^, ^97^, ^98^, ^99^ ^]^ enzymatic crosslinking,^[^ ^95^, ^100^ ^]^ self‐assembly,^[^ ^101^ ^]^ and hydrogen bonding^[^ ^102^ ^]^ also exhibit similar shear‐thinning behavior, allowing easy extrusion of the ink from the nozzle. However, due to the highly dynamic nature of supramolecular interactions, the 3D printed objects may lack structural integrity and can fuse together or disperse in aqueous conditions, compromising the resolution and 3D printability.^[^ ^94^ ^]^ Introducing both covalent and noncovalent networks into hydrogel systems has been exploited as a highly efficient strategy to significantly enhance the printability of hydrogels by DIW. The use of a noncovalent crosslinkers allows inks to flow and extrude under low shear and have immediate shape retention postdeposition. The shape stability of the printed features could be significantly enhanced through a second step of covalent crosslinking. For example, through photoinitiated covalent crosslinking,^[^ ^86^, ^91^, ^94^, ^95^, ^102^, ^103^, ^104^, ^105^ ^]^strong ionic interactions of alginate with calcium,^[^ ^92^, ^101^, ^106^ ^]^ or horseradish peroxidase/hydrogen peroxide crosslinking (H_2_O_2_).^[^ ^95^ ^]^ Burdick and co‐workers reported that despite achieving 3D layer‐by‐layer grid structures using hydrogels crosslinked with only a supramolecular network, the filaments were still fused together when printing multilayers due to the dynamic nature of supramolecular interactions (Figure 3D). Due to the strong covalent bond crosslinking, the 3D printed structures could not be well defined . In contrast, excellent printability was found in the multilayered 3D structures printed from dual crosslinked hydrogels because the robust covalent crosslinking network could stabilize printed structures against the strain of the relaxation of the supramolecular network.

Besides UV induced free radical polymerization, the photoinitiated thiol−ene,^[^ ^107^ ^]^thiol‐yne click chemistry^[^ ^108^ ^]^ and thiol‐Michael reactions,^[^ ^109^ ^]^ which can form a more homogeneous (step‐growth) hydrogel network^[^ ^110^ ^]^ compared to that of a free radical polymerized diacrylates were utilized for post‐crosslinking after extrusion.^[^ ^106^ ^]^ The strong metal coordination bond between Fe^3+^ and COOH^−^ was also employed to encourage fast gelation of the highly viscous linear polymer solutions after extrusion,^[^ ^39^, ^111^ ^]^ which significantly simplified the 3D printing process of hydrogels.

To overcome the difficulty and complexity of the dual‐network hydrogels systems, Hong and co‐workers designed and synthesized a novel type of single‐network hydrogel for 3D printing.^[^ ^112^ ^]^ Such a system requires only one light stimulus to initiate gelation. After the acrylated polycaprolactone−poly(ethylene glycol)−polycaprolactone precursors with controlled viscosity were extruded, visible light was utilized to irradiate the polymers to efficiently trigger gelation. Visible light was used for crosslinking rather than the more common UV crosslinking (365 nm), making this safer for biological applications. Interestingly, without the need for post‐printing treatment to enhance the dimensional stability, the 3D printed multilayer structures from hydrogels formed by DNA hybridization^[^ ^113^ ^]^ and electrostatic interactions^[^ ^93^ ^]^ exhibited high viscosity and showed no obvious shrinking or swelling phenomena upon subsequent printing of multilayers.

Although shear‐thinning behavior was also found with covalently crosslinked hydrogels systems, it is still a huge challenge to print covalently crosslinked gels by DIW due to the higher strength of covalent bonds. The adjustment and optimization of ink formulations should be carefully executed through precise control of the polymer concentration and degrees of crosslinking.^[^ ^86^, ^114^, ^115^ ^]^ There is a clear operational window for DIW printing: the gels should not be too soft and spreadable (low concentration and crosslinking density) nor too stiff to be extruded (high concentration and crosslinking density). In contrast to physically crosslinked hydrogels, the use of dynamic covalent crosslinked hydrogels as the ink enabled the formation of well‐structured and mechanical stable filamentous strands.^[^ ^114^, ^115^ ^]^ Subsequent printing of multiple ink layers on top of each other does not cause spreading or collapsing of the network.

Incorporating inorganic nanomaterials represents another simple but efficient method to improve the 3D printability of hydrogels. For example, Laponite nanoclay is one of the most studied additives used to enhance the 3D printability of hydrogels.^[^ ^116^, ^117^, ^118^ ^]^ This material is a type of hydrous sodium lithium magnesium silicate that consists of nanoscale platelets/disks with both positive and negative charges. The presence of charges and functional hydroxyl groups on the surface of the Laponite nanoclay results in a very strong interaction with polymers. Huang and co‐workers have studied the effect of the Laponite nanoclay on the rheological properties, including the shear moduli, yield stress, and thixotropic time of the NIPAAm hydrogels.^[^ ^117^ ^]^ It was demonstrated that the addition of Laponite nanoclay resulted in a much higher storage modulus than the loss modulus of both NIPAAm–Laponite nanocomposites, indicating a solid‐like behavior in a sheared condition and better printability. The yield stress of the nanocomposite (≈100 Pa) was found to be much higher than pure PNIPAM, indicating excellent self‐supporting properties. The viscosity of the nanocomposite hydrogel undergoes a significant and rapid change under a shearing force. Other inorganic nanomaterials with thixotropic properties such as kappa‐carrageenan, nanosilicates,^[^ ^119^ ^]^ and cellulose^[^ ^120^, ^121^, ^122^ ^]^ have also been utilized to tune the rheological properties of hydrogels to enhance their printability.

Besides inorganic filters, carbomer, which is a crosslinked high‐molecular weight polymer of acrylic acid, was proposed as a new type of highly efficient rheology modifier for printing different types of hydrogels including double network (DN) hydrogels, nanoparticle reinforced functional hydrogels, stimuli‐responsive hydrogels and common hydrogels (Figure 3E).^[^ ^87^ ^]^ The mechanism of carbomer as rheology modifiers could be interpreted as the formation of microgel structures which could resist shear stress due to ionization of carboxyl groups. In addition, gelatin was also reported to induce the sol–gel transition of polyurethane (PU) aqueous solution.^[^ ^123^ ^]^ Due to the significantly increased viscosity and storage modulus of the bio‐ink, excellent stacking ability (up to 80 layers) and high‐resolution printing (through an 80 µm nozzle) were successfully realized.

### 2.3 Other Polymers

Polydimethylsiloxane (PDMS) is a very interesting class of elastomer and has attracted extensive attention in a wide range of applications due to its good material elasticity, high elongation, biocompatibility, low cost, and transparency.^[^ ^124^ ^]^ It has been widely used for fabricating functional micro–nano devices by photolithography,^[^ ^125^ ^]^ soft lithography^[^ ^126^ ^]^ or block copolymer thin film assembly.^[^ ^127^ ^]^ With the recent advances in 3D printing, the fabrication of complex 3D shapes from PDMS has been reported.^[^ ^128^ ^]^ Lau and co‐workers studied the printability of PDMS by the DIW method as a function of incorporated nanosilica into the PDMS matrix.^[^ ^129^ ^]^ The oscillatory shear test shows that the extruded filaments from pure PDMS quickly spread on the substrate upon exiting the nozzle and the desired 3D layer‐by‐layer patterns could not be achieved, due to the liquid‐like behavior in the printing process. The high surface area silica particles could not only transform the pure Newtonian PDMS fluid into shear‐thinning fluid, but also prevent the printed pattern (ink) from collapsing by enhancing the mechanical strength. However, high silica loading (above 20 wt%) could result in extremely high viscosity (2600 Pa s at a shear rate of 2 s^−1^) and nanoparticle aggregation which may clog the micronozzle. On the other hand, a low silica loading may cause significant collapse of the printed structure. Since the PDMS‐based materials typically exhibit poor temperature stability beyond −50 °C due to a crystallization transition occurring at −75 °C, the sterically hindered diphenyl moieties were incorporated into PDMS chains to achieve better low temperature stability and 3D printability.^[^ ^130^ ^]^ The extrudable PDMS‐based ink could also be achieved by blending uncured PDMS liquid precursor with PDMS microbeads, due to capillary attraction induced by the liquid precursor.^[^ ^131^ ^]^ Dynamic oscillatory measurements reveals that inks containing certain fractions of PDMS liquid precursor behaved like pastes, which are flowable at high shear stress and possess high storage moduli and yield stresses that are desirable for DIW. Advincula and co‐workers have demonstrated that the incorporation of salt gel in the ink could transform the liquid like PDMS resin (loss modulus > storage modulus) into a thixotropic gel‐like ink (storage modulus > loss modulus).^[^ ^132^ ^]^ This group also realized the 3D printing of polyurethane suspension through the addition of fillers including nanoclay and Si nanoparticles.^[^ ^133^ ^]^ In order to enhance the ink stability after extrusion, dibutyl phthalate (DBP) was added to promote the adhesion strength between individual layers.

Gracias and co‐workers recently proposed a new approach for 3D‐printing polytetrafluoroethylene (PTFE) which has high melting viscosity using DIW (Figure 3F).^[^ ^88^ ^]^ They formulated the surfactant‐stabilized PTFE nanoparticles ink with a binding gum which can provide shear‐thinning property. A thermal treatment was used after printing to coalesce PTFE nanoparticles, to form the final structure, and to remove additives. This also overcomes the poor printability due to rapid crystallization and weak adhesion of commercially available poly(ethylene terephthalate) PET.

The advantage of DIW systems includes no toxic vapor generation as opposed to FFF 3D printing. DIW produced materials are significantly softer (kPa range) compared to FFF produced materials (MPa or GPa range) and fine tuning of ink rheology is usually required which can narrow the operational window of these systems. Future DIW materials could be developed to be stronger and tougher to be applicable for a wider range of applications such as engineering and construction.

## 3 Retaining Material Properties of Printed Polymers

It is important to study and understand if the material properties can be retained after being printed and how the 3D printed structures affect the final device performance. Compared to traditional microfabrication technologies, 3D printing allows direct fabrication of materials into 3D geometries as well as combining diverse functional materials using a single printing tool. It is thus expected that the 3D printed object would exhibit enhanced properties. In this section, we will first present different materials properties of printed polymers, specifically mechanical, electrical and integrated properties. Subsequently, the design of stimuli responsiveness into 3D printed polymeric materials including shape morphing, degradability and color changing is discussed.

### 3.1 Engineered Mechanical Properties

High mechanical strength is one of the most essential material properties and is particularly important for some key fields such as artificial muscles, medical devices, load bearing soft tissue replacements, soft robots, and biomimetic applications. However, most printable polymers suffer from very poor mechanical properties, thus limiting their applications. In response to such a challenge, tremendous efforts have recently been devoted to developing novel polymers with high strength and toughness including double‐network strategy,^[^ ^134^, ^135^, ^136^, ^137^ ^]^ incorporating nanocomposites,^[^ ^138^, ^139^, ^140^ ^]^ modulating topography,^[^ ^141^ ^]^ and various noncovalent bonds with a wide spectrum of bonding strength.^[^ ^39^, ^142^, ^143^, ^144^, ^145^, ^146^ ^]^ It is extremely challenging to use 3D printing to process these hydrogels due to their toughness which makes them unextrudable or require excessive pressure to facilitate flow.

**Figure 4:**
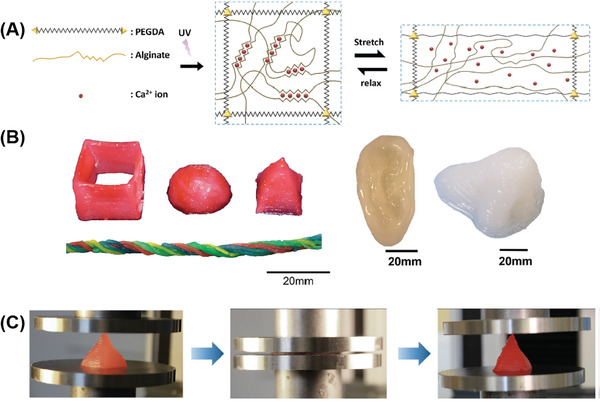
An example of good mechanical properties of a 3D printed double network polymers. A) Schematic diagrams of the tough double‐network hydrogel, ionically crosslinked alginate with of Ca^2+^ and covalently crosslinked poly (ethylene glycol) (PEG). Reproduced with permission.^[^ ^147^ ^]^ Copyright 2015, John Wiley and Sons. B) Various 3D constructs printed with the double network hydrogel. C) A printed pyramid undergoes a compressive strain of 95% while returning to its original shape after relaxation.

The ionic crosslinking between Ca^2+^ and alginate is one of the most widely used strategies for endowing hydrogels with high toughness and strength.^[^ ^135^ ^]^ Tough hydrogels consisting of Ca^2+^ crosslinked with alginate and covalently crosslinked poly (ethylene glycol) (PEG) have been 3D printed by Zhao and co‐workers^[^ ^147^ ^]^ The authors ascribed the high stretchability and toughening mechanism as the dissipation of mechanical energy by reversible Ca^2+^ crosslinking while maintaining the elasticity from covalently crosslinked PEG (**Figure** **4**A). The 3D printability of the tough hydrogel was achieved through controlling the viscosity of the pre‐gel solution by adding biocompatible nanoclay. Not only was a diverse range of 3D shapes such as a hollow cube, hemisphere, pyramid, twisted bundle, and human nose and ear models created, but the printed objects were made from multiple materials (Figure 4B). Their printed hydrogel structures are highly stretchable and tough. They reported a printed pyramid that underwent a 95% compressive strain but still regained 97% of its original height after unloading (Figure 4C). Cyclic stress–strain studies showed that the hysteresis in the first loading–unloading cycle, indicated that the hydrogel could effectively dissipate the energy. The observed retention of fracture toughness after deformation suggests excellent antifatigue resistance. The Spinks's group demonstrated 3D printing of Ca^2+^ crosslinked double network alginate–acrylamide hydrogels, by using the modified extrusion system.^[^ ^148^ ^]^ The alginate–acrylamide pre‐gel inks were lightly crosslinked with Ca^2+^ before printing to facilitate extrusion and structural stability, while UV irradiation was then employed to induce the second hydrogel network. The dog bone specimens were successfully printed after carefully optimizing the concentrations of inks. The tensile testing results show that the printed hydrogels exhibited remarkable mechanical performance with Young's modulus, failure stress, and failure strain as high as 83, 170 kPa, and 300%, respectively.

Using oppositely charged electrostatic interactions is another attractive and novel strategy to construct tough hydrogels and was proposed by Gong and co‐workers in 2013.^[^ ^143^ ^]^ These hydrogels, which are copolymers of sodium *p*‐styrenesulphonate (NaSS) and 3‐(methacryloylamino)propyl‐trimethylammonium chloride (MPTC),could be changed into transparent viscous solutions in the presence of a high sodium chloride (NaCl) concentration, while observing rapid recovery to the opaque tough hydrogels after removing the salt. Utilizing such properties of polyion complex (PIC) hydrogels, Wu and co‐workers reported 3D printing the hydrogels based on poly(3‐(methacryloylamino)‐propyl‐trimethylammonium chloride) and poly(sodium *p*‐styrenesulfonate) (PMPTC and PNaSS).^[^ ^149^ ^]^ Before 3D printing, the charged hydrogels were plasticized into a viscous solution by saline water. The rheological behavior of the viscous solution was optimized by tuning the temperature and ratio of hydrogel to salt. The extruded viscous solution was then treated with a pure water solution which lead to the rapid gelation and stabilization of printed structures. In water, the viscous PIC solution rapidly gelated into a tough solid gel by dialyzing out the salt and counterions of PIC. The 3D printed hydrogels exhibited excellent mechanical properties with the measured Young's modulus (*E*), tensile strength (*σb*), and breaking strain (*εb*) of the printed fiber were 2.6, 1.2 MPa, and 530%, respectively. Complex 3D structures with high shape fidelity from nanocomposite hydrogels have also been fabricated. 3D printing of DN hydrogels with a low‐cost printer was also demonstrated.^[^ ^150^ ^]^ The ink was formulated with a layered silicate rheology modifier, a photoinitiator (Irgacure D‐2959), sodium 2‐acrylamido‐2‐methylpropanesulfonate (AMPS) and *N*,*N*‐methylenebis(acrylamide) (MBAA). achieve shape stability after extrusion without the frequent clogging of the nozzle, the content of Laponite as well as the concentration of the monomers were optimized to achieve appropriate viscosity. Excellent mechanical properties were also demonstrated with the maximum compression strength, elastic modulus, elongation strain and toughness of 61.9, 0.44 MPa, and 923%, 3024 kJ, respectively. 3D printed hydrogels based on graphene oxide (GO),^[^ ^151^ ^]^ carrageenan and nanosilicates^[^ ^119^ ^]^ as well as cellulose^[^ ^120^, ^121^ ^]^ all showed outstanding mechanical properties. Suo and co‐workers printed 3D multilayer structures of a hydrogel and an elastomer in arbitrary sequence. The precursors of elastomer ink contain the photoinitiator, which enables the formation of interlinking polymer network upon curing process.^[^ ^152^ ^]^ Such 3D printed hydrogel/elastomer hybrid device exhibits high fracture energies (≈10 000 J m^−2^ for the hydrogel and ≈6000 J m^−2^ for the elastomer) as well as high adhesion energy (≈5000 J m^−2^).

3D printed devices from thermoset polymers can exhibit excellent mechanical properties, which is very promising for high‐performance applications, such as mechanical parts and aerospace. However, many great challenges remain, such as lower printing speeds, poor and anisotropic mechanical properties. In response to such challenges, Qi and co‐workers developed a novel type of thermoset epoxy composite ink composed of a UV curable resin and an epoxy oligomer.^[^ ^153^ ^]^ During the 3D printing process, a single layer of epoxy filament was deposited on the platform through DIW, which was then cured by UV irradiation for the purpose of maintaining the desired 3D printed shape at a high temperature. The printed objects then underwent a second step of thermal curing to facilitate the formation of an interpenetrating polymer network through epoxy oligomer polymerization. The tensile stress–strain curves of the printed samples with different printing paths were similar, indicating that the Young's modulus and tensile strength of printed epoxy composites were insensitive to the printing path. Besides isotropic mechanical properties, such printed epoxy composites show outstanding tensile toughness with a maximum Young's modulus of 1.31 GPa, an ultimate strain of 32.1% and tensile strength of 7 MPa. The isotropic mechanical properties of 3D printed thermoset polymers could also be achieved through incorporating dynamic covalent crosslinker between layers in printed parts. Voit and co‐workers applied reversible furan‐maleimide Diels–Alder chemistry to construct printable thermoset resins with isotropic and excellent mechanical properties.^[^ ^61^ ^]^ It was found that all the printed layers were fused into each other due to dynamic reversible Diels–Alder chemistries, which results in isotropic mechanical properties with toughness of 18.59 ± 0.91 and 18.36 ± 0.57 MJ m^−3^ perpendicular and parallel to the build direction.

**Figure 5:**
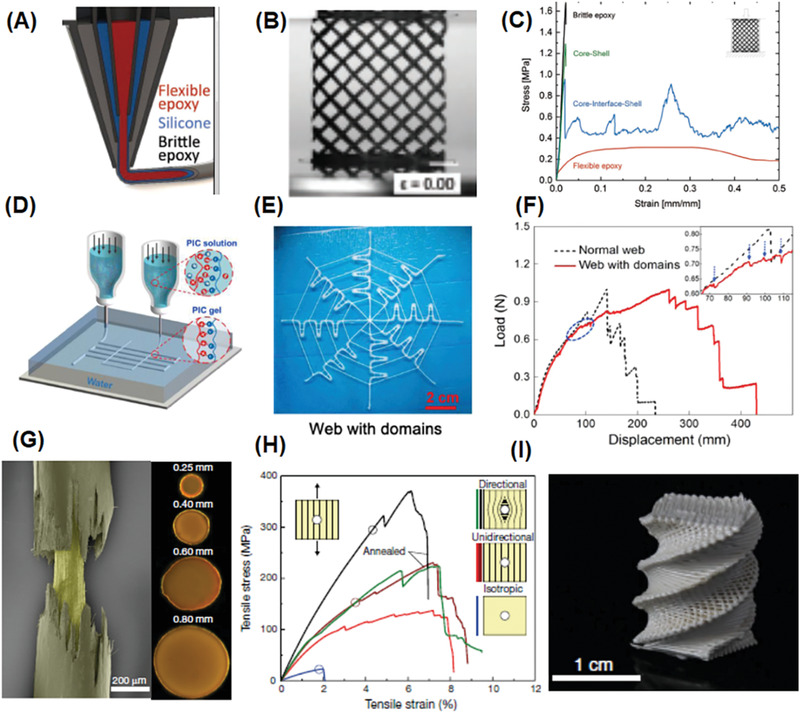
The mechanical properties of 3D printed polymers realized through engineering complex material architectures. A–C) 3D printed core–shell epoxy composite. Reproduced with permission.^[^ ^154^ ^]^ Copyright 2019, John Wiley and Sons. A) Schematic cross‐sectional view of core–shell printing technique. B) Optical images and C) stress–strain curves of the fabricated 3D core–interface–shell lattice showing tunable properties from the complex structure. D–F) 3D printed hydrogel mimicking titin structure. Adapted with permission.^[^ ^155^ ^]^ Copyright 2018, American Chemical Society. D) Customized 3D printing system with multiple syringes used to print the polyion complex hydrogels. E) Photo of printed network structures consisted of well‐defined thick and thin fibers mimicking spider‐webs. F) Snapshots at certain displacement and force–displacement curves of the printed structure showing improved properties due to the web structure. G–I) 3D printed aromatic thermotropic polyesters. Adapted with permission.^[^ ^156^ ^]^ Copyright 2019, Nature Publishing Group. G) Characterizing core–shell structure of the extruded liquid crystal polymer filaments: False‐colored scanning electron microscopy (left) and polarized light microscopy (right) of the extruded filaments. H) Mechanical properties of printed liquid crystal polymers with well‐designed 3D filament architecture, demonstrating the influence of morphology on properties. I) An example of 3D‐printed liquid crystal polymer parts with complex fiber architectures and geometries.

Utilizing the excellent design freedom provided by 3D printing, unprecedented mechanical properties could be realized through engineering complex material architectures. Inspired by natural materials such as bone, which possesses both high stiffness and toughness due to combining toughening mechanisms across multiple length scales, Lewis and co‐workers realized both high stiffness and toughness in the 3D printed epoxy polymers with complex lattices architectures, through constructing flexible epoxy core–brittle epoxy shell motifs with an elastomeric silicone interfacial layer.^[^ ^154^ ^]^ The core–shell complex structures (**Figure** **5**B) with different mechanical properties were fabricated through coextrusion of coaxially aligned epoxy and silicone resins via designed multicore–shell printheads (Figure 5A). The stiffness and breaking strength of the individual composite was measured to be 1.25 GPa and 22 MPa, respectively. The superior energy absorption capabilities and toughness were demonstrated when the material was printed into the architected lattice and subjected to compression: the shells fractured into pieces while the core remained intact (Figure 5C). Furthermore, Wu and co‐workers fabricated hierarchical structures that consisted of polyion complex hydrogels with different diameters using multiple nozzles (Figure 5D–F).^[^ ^155^ ^]^ It was demonstrated that thick and thin hydrogel fibers exhibited similar breaking elongation, but quite different load‐bearing abilities.

Taking advantage of 3D printing free design, heterogeneous hydrogel structures were successfully fabricated, which consisted of different weak (thin) or tough (thick) materials at selective locations (Figure 5E). When stretched, the weak portion broke first, while the tough fibers prevented the failure of the main structures, mimicking the toughening principle in titin. When such structures are incorporated into a spider web structure, it exhibits extremely high extensibility and toughness, which are far better than a normal spider web (Figure 5F). The ability of liquid crystals to assemble into a highly oriented domains with hierarchical architectures and complex geometries has been utilized during extrusion based 3D printing.^[^ ^156^ ^]^ After extrusion, the core–shell structure of the aromatic thermotropic polyesters spontaneously formed (Figure 5G) due to the formed temperature gradient between the cold surface of the filament and its hot interior. The produced core–shell filaments showed notable mechanical strength and elastic modulus. Moreover, because the liquid crystal mesogens were oriented along with the printing path, such printed anisotropic liquid crystal polymers devices exhibited high stiffness, strength and toughness that outperforms state‐of‐the‐art 3D‐printed polymers by an order of magnitude (Figure 5H).

### 3.2 Electrical Properties

Polymer electronics is an emerging technology that incorporates electrically conductive and semiconducting polymeric materials to develop electronic devices. These soft electronic devices combine the electronic properties of the polymers with good processability. In this section, we give a short summary of electrical performance of various 3D printed polymeric electronics, including electrodes, strain sensors, polymer electrolyte, photodetectors and nanogenerators.

#### 3.2.1 Battery Electrodes

**Figure 6:**
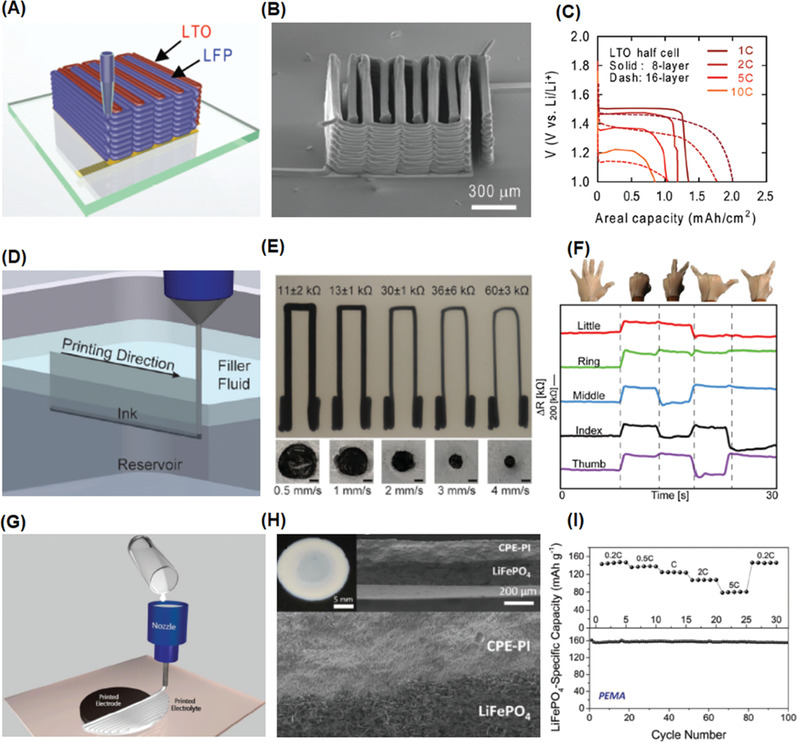
Methods and outcomes of 3D extrusion printing of polymeric electronic devices. A) Schematic illustration of 3D printed microbattery. Reproduced with permission.^[^ ^158^ ^]^ Copyright 2014, John Wiley and Sons. B) SEM images of printed and annealed 16‐layer interdigitated electrode architectures. C) Comparison of the energy and power densities of printed, 3D interdigitated microbattery architectures (3D‐IMA) to reported literature values. D) Schematic illustration of the embedded 3D printing process. Reproduced with permission.^[^ ^160^ ^]^ Copyright 2014, John Wiley and Sons. A conductive ink is printed into an uncured elastomeric reservoir, which is capped by filler fluid. E) Top and cross‐sectional images of soft sensors printed at varying speeds. F) Electrical resistance change as a function of time for printed strain sensors within the glove at five different hand positions. G) Schematic illustration of the filamentary printing process for fabricating poly(vinyldene fluoride)‐based electrolyte. Reproduced with permission.^[^ ^161^ ^]^ Copyright 2014, John Wiley and Sons. H) Photograph and cross sectional SEM images. I) Rate performance (top) and cycling performance of a fully printed poly(vinyldene fluoride)‐based electrolyte.

3D printing technology with the ability to arbitrarily define the micro‐ and nanostructured architectures^[^ ^157^ ^]^ has emerged as a simple and versatile approach to fabricate electrodes with precisely controlled architectures. This approach can lead to a better ion diffusion. 3D printed microscopic architectures could also play an important role in the electrical performance which is superior to microporous device counterparts fabricated by traditional methods. Lewis and co‐workers pioneered 3D printing of Li‐ion battery electrodes.^[^ ^158^ ^]^ They prepared the Li_4_Ti_5_O_12_ (LTO) anode and LiFePO_4_ (LFP) cathode ink formulated with cellulose‐based viscosifiers. High aspect ratio (from ≈0.8 to 11) multilayer electrodes were printed onto a glass substrate through a cylindrical nozzle (**Figure** **6**A,B). The 16 layer 3D microbattery architectures exhibit a high areal energy density of 9.7 J cm^−2^ at a power density of 2.7 mW cm^−2^ (Figure 6C), which is superior to other rechargeable microbattery counterparts reported in the literature. Such excellent performance could be ascribed to the fabrication of 3D printed high‐aspect structures that occupy a small footprint and maintain small transport length scales to facilitate ion and electron transport during charging and discharging processes. In order to improve the low electrical conductivity (10^−4^–10^−6^ S cm^−1^) reported in this study, Hu and co‐workers fabricated all components in their 3D‐printed lithium‐ion batteries through layer‐by‐layer printing of GO‐based electrodes which possess good electrical conductivity,^[^ ^159^ ^]^ with poly(vinylidenefluoride)‐*co*‐hexafluoropropylene (PVDF‐*co*‐HFP) as solid‐state electrolyte. The additional advantages of adding GO included excellent shear‐thinning behavior, ink stability over relatively long time periods, as well as good loss moduli stability. The electrical conductivities of LFP/reduced graphene oxide (rGO) and LTO/rGO electrodes were measured to be 31.6 and 6.1 S cm^−1^, respectively, which significantly improved the results of the previous study. The 3D printed fuel cell also exhibits a reversible capacity of 91 mA h g^−1^.

Yang and co‐workers demonstrated the first example of a 3D printed sulfur–copolymer–graphene electrode for Li–S batteries using ink composed of sulfur particles, 1,3‐diisopropenylbenzene (DIB), and condensed graphene oxide dispersion.^[^ ^162^ ^]^ Due to the presence of concentrated graphene oxide, the ink exhibited a high elastic modulus (>104 Pa) and shear yield stress (>102 Pa) making it suitable for 3D printing. The 3D microlattices with a center spacing of 800 µm and bar diameter of 200 µm were printed layer‐by‐layer onto a substrate, which was subsequently heated to facilitate the formation of sulfur copolymers on the surface of the reduced graphene oxide via facile copolymerization. The 3D printed Li–S batteries exhibits a high reversible capacity of 812.8 mA h g^−1^ and a good cycle performance, which could be attributed to the suppressed polysulfides dissolving by sulfur copolymers, as well as high conductivity from GO. Zhu and co‐workers reported 3D printing of graphene aerogel (GA)‐based electrodes with polypyrrole (PPy)^[^ ^163^ ^]^ which is one of the most widely used pseudocapacitive materials and could boost specific capacitance of graphene through synergetic effects. Hydroxypropyl methylcellulose (HPMC) solution was added into ink formulation as a viscosifier to impart both shear thinning behavior and a higher yield stress. An amphiphilic triblock copolymer pluronic F127 was used not only to induce viscoelastic properties but also act as a soft template. The woodpile cubic lattices, consisting of multiple orthogonal layers of parallel cylindrical filaments, was printed with excellent structural integrity and accuracy. The specific gravimetric capacitance of the 3D printed electrode was measured as high as 395 F g^−1^ with 90% retention after 5000 charge–discharge cycles. These printed electrodes also have very strong compressive strength (2.4 MPa). Areal capacitance and energy density of the PPy–GA electrode were measured to be 2 F cm^−2^ and 0.78 mWh cm^−2^, respectively, which represent the current gold standard for compressible electrodes. Using a similar strategy, the electrodes based on carbon nanotubes were also printed using a composite ink composed of silver nanoparticles and silver flakes and a thermoplastic polystyrene–polyisoprene–polystyrene (SIS) triblock copolymers.^[^ ^164^ ^]^ The thermoplastic polymer was used to enhance shear thinning behavior. The high conductivity of the printed electrodes was easily obtained after post‐treatment at a temperature as low as 80 °C, without additional processing. This could be ascribed to the presence of positively charged amine‐functionalized carbon nanotubes and negatively‐charged carboxyl‐terminated silver nanoparticles.

#### 3.2.2 Soft Strain Sensors

Strain sensors are an essential component of soft electronics and have recently attracted considerable attention in diverse fields, for example as devices for health‐monitoring, artificial skin and functionalized prosthetic limbs. The sensors are composed of a deformable conducting material placed onto an inactive stretchable material.^[^ ^165^ ^]^ The soft strain sensors are capable of translating the mechanical signal into an electrical signal through several mechanisms, including a geometrical effect, piezo‐resistive effect, disconnection mechanism, crack propagation, and tunneling effects. 3D printing is an attractive method for fabricating strain sensors due to its key advantages compared to traditional fabrication technologies, such as a lower cost, large manufacturing scalability, high resolution, and outstanding structural flexibility. In 2014, the Lewis Group pioneered the work of fabricating strain sensors by the DIW method (Figure 6D).^[^ ^160^ ^]^ The ink was formulated by homogenizing the as‐received carbon conductive grease, which exhibits a strong shear thinning response. However, void space defects were found after the viscoelastic ink was directly extruded into the underlying elastomeric substrate. To prevent the underlying substrate from breaking up, a thinning agent was added into a commercially available silicone elastomer Ecoflex 00–30. The rheological properties of the substrate are important, as the storage modulus needs to be high enough to support the patterned ink filaments without distorting their geometry. The hairpin (U‐shape) configuration composed of a cylindrical wire‐like features, truncated with large contact pads at each end, was fabricated on the Ecoflex soft substrate (Figure 6E). The embedded strain sensors were demonstrated to not only have large extensibility (up to 900% elongation) and softness, but they were also able to monitor digit movement of a user's hand through electrical resistance changes as a function of time (Figure 6F). In another example, the same authors developed conductive inks based on a thermoplastic polyurethane (TPU) and silver flakes.^[^ ^166^ ^]^ 3D functional strain and pressure sensor inks consisting of insulating matrix and conductive electrode were printed onto specific layouts through separate DIW. Such hybrid methods enable complex microcontroller devices coupled with a strain sensor and a large‐area soft sensor array, which could be utilized to monitor the joint bending movements along with the corresponding LED readout.

Ionically conductive hydrogels have shown great potential in the field of soft strain sensors^[^ ^167^ ^]^ due to their high stretchability, transparency, softness, biocompatibility, and facile synthesis. Wu and co‐workers prepared interesting thermoresponsive hydrogels with controlled volume phase transition temperatures at ≈30 °C, close to the human body temperature.^[^ ^168^ ^]^ Such physically crosslinked hydrogels are suitable for 3D printing and enables fabricating sub‐millimeter resolution grid‐structured hydrogel films. In the presence of salt solutes, it was found that this 3D printed hydrogel sensor could amplify pressure sensitivity due to the well‐controlled microscale structures, which allows the devices to sense body temperature and human motion. Using 3D extrusion printing, Vlassak's group fabricated a sub‐millimeter resolution device with an ionically conductive PAAm hydrogel and a PDMS dielectric elastomer.^[^ ^169^ ^]^ The printed hybrid devices were then attached to the index finger of a nitrile glove and connected to a multimeter to measure the resistance of the sensor. The printed devices are sensitive enough to detect inadvertent finger motions of the ring finger in position two, as well as intermediate positions between being fully bent and fully straightened. 3D extrusion technology also enables direct printing of highly conductive poly(ethylene oxide) based hydrogels onto human hands to visually track hand movement,^[^ ^170^ ^]^ as well as live mice, providing a unique opportunity to study wound‐healing capabilities.

#### 3.2.3 Polymer Electrolyte

Solid‐state polymer electrolytes are a good candidate to replace liquid electrolytes because of the balanced electrochemical and mechanical properties, plus safer handling/storage.^[^ ^171^ ^]^ The high viscosities and low melting points of polymer‐based electrolytes makes them ideal candidates for 3D extrusion. 3D extrusion printing enables a well‐controlled tight and continuous interface between the electrode and electrolyte layers, which is desirable for discharge voltage stability in a flexible energy storage device under mechanical stress. Durstock and co‐workers developed high‐performance, flexible, and printable polymer electrolytes for Li‐ion batteries (Figure 6G).^[^ ^161^ ^]^ The ink was formulated with PVDF and a nanosized Al_2_O_3_ filler, which was further treated by a drying step to generate sub‐micrometer pores. During the printing process, some of the electrolyte ink could diffuse into the porous composite electrode leading to excellent interfacial adhesion (Figure 6H). The printed electrolyte demonstrates high‐rate electrochemical performance (Figure 6I) but possesses better wetting characteristics and enhanced thermal stability. In order to simplify the electrolyte fabrication process, elevated temperature DIW techniques were developed for directly printing the ink onto the electrode.^[^ ^172^ ^]^ A composite ink composed of poly(vinylidenefluoride‐hexafluoropropylene) matrices, Li+ conducting ionic‐liquid electrolyte and nanosized ceramic fillers were developed. The 3D printing could facilitate the formation of a dense layer between the porous polymer‐based electrolyte and the electrode, which was found to significantly reduce the interfacial resistance. As a result, the batteries with the printed electrolytes showed higher charge/discharge capacity values and a better performance than the batteries where the electrolyte was fabricated by traditional methods.

#### 3.2.4 Other Soft Electrical Devices

Triboelectric nanogenerator (TENG) devices which could generate electricity by triboelectrification and electrostatic induction have also been printed into 3D devices.^[^ ^173^ ^]^ You and co‐workers utilized 3D extrusion printing to fabricate the triboelectric nanogenerator with poly(glycerol sebacate) (PGS) and carbon nanotubes (CNTs) as the two electrification components.^[^ ^174^ ^]^ Due to the hierarchical porous structure of the 3D printed device, the device output efficiency was demonstrated to be much superior to traditional molded microporous TENG counterparts. Recently Zhang and co‐workers reported direct printing of core‐sheath fiber‐based smart patterns for energy/management E‐textile, using CNTs as a conductive core and silk fibroin as a dielectric sheath.^[^ ^175^ ^]^ Chen and co‐workers demonstrated that it was possible to fabricate the asymmetric GO/polyaniline (PANi) microsupercapacitors using 3D extrusion printing which exhibits improvement in the voltage window, energy density, power density, and cycling stability compared with the symmetric microsupercapacitors.^[^ ^176^ ^]^ Photodetectors consisting of semiconducting polymer poly(3‐hexylthiophene) (P3HT):[6,6]‐phenyl C61‐butyric acid methyl ester (PCBM) blend were printed via a layer‐by‐layer deposition process. Such printed devices showed an external quantum efficiency of 25.3%, which is comparable to that of microfabricated counterparts.^[^ ^177^ ^]^ The 3D printing approach enables photodetectors to be fabricated directly on flexible substrates and hemispherical surfaces, which is more promising in real‐life applications.

### 3.3 Integrated Properties

Polymeric materials with increasingly sophisticated properties and multiple functions have become emerging areas of research.^[^ ^178^, ^179^, ^180^ ^]^ This includes the design of polymer materials that can possess complex and often counter‐intuitive properties. For example, self‐healing materials rarely have high strength. Combining these types of properties into the one material greatly expands their scope for new applications, as many applications benefit from combinations of advanced properties, such as self‐healing^[^ ^42^ ^]^ high conductivity,^[^ ^181^, ^182^ ^]^good mechanical performances (toughness, stretchability, self‐recovery),^[^ ^183^, ^184^, ^185^ ^]^ stimuli‐responsiveness,^[^ ^186^ ^]^ biocompatibility,^[^ ^187^ ^]^ and reprocessability.^[^ ^60^, ^178^ ^]^

#### 3.3.1 Mechanically Strong and Biocompatible Polymers

To make 3D printed polymer devices suitable for biological applications, the polymers should be biocompatible for guiding cell growth and tissue generation. Additionally, they need to be mechanically strong to prevent damage to the scaffold. The rapid development of 3D printing technologies, especially extrusion methods, provides an attractive opportunity to produce high performance 3D growth‐directing structures with both good biological and mechanical properties to mimic the functions of native tissues. Advincula and co‐workers demonstrated FFF 3D‐printing of TPU/PLA/GO nanocomposites.^[^ ^55^ ^]^ Both PLA and TPU have been commonly used in biomedical applications because of their high biocompatibility. However, TPU only showed moderate tensile strength despite high elongation, while PLA is very brittle although it is mechanically strong. Blending of PLA and TPU could effectively enhance the overall material properties, where further addition of GO could contribute to improving the compression strength, thermal stability and cell growth. The 3D printed microlattice showed a high compression and tensile modulus, which are 107.21 and 90.02 MPa, respectively. Furthermore, the NIH3T3 mouse embryonic fibroblast cells were successfully seeded on the 3D printed TPU/PLA GO monolayers. The live/dead assay results indicate that only live cells are detected on the printed scaffold support, while no dead cells were detected on any scaffold.

Hydrogels are often intrinsically biocompatible due to their structural similarity to the natural extracellular matrix (ECM). However, a key challenge is to increase their mechanical properties in order to expand their uses in biological applications. Chitosan hydrogels, which contain an ionizable amino group, have been developed as the ink for 3D extrusion printing by dissolving them in acidic solvents.^[^ ^102^ ^]^ The thermal responsiveness of the chitosan polymers can be induced with modification of the hydroxybutyl group, resulting a rapid sol−gel transition. After the polymer is extruded from the micronozzle, the printed chitosan undergoes a solidification process due to solvent evaporation, which is then further treated with a basic solvent to trigger gelation via hydrophobic interactions and hydrogen bonding. The printed filaments are not only flexible but also exhibited high strain at failure (≈400%) and ultimate strength (≈7.5 MPa). The chitosan scaffolds showed full coverage of live L929 fibroblasts with only very few or no dead cells. The printability and mechanical properties of chitosan could also be enhanced upon the addition of salt.^[^ ^91^ ^]^ After treating the 3D printed hydrogel constructs with a 10% NaCl solution, excellent mechanical properties with stress and elastic modulus as high as 5.6 and 48.0 MPa, were observed respectively. Good elastic recovery performance of the 3D‐printed scaffolds was demonstrated, with the polymer being able to quickly recover to its original state without distortion after substantial deformation. Its excellent biocompatibility was also shown, with high cell viability observed on the grids after 7 days of culturing chondrocytes on the scaffolds.

**Figure 7:**
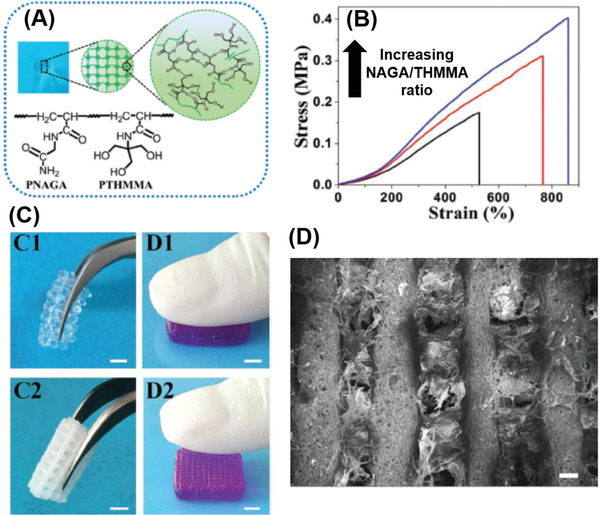
3D printed hydrogels, their mechanical properties and biocompatibility. A) Molecular structure and hydrogen bonding interactions. B) Tensile stress–strain curves of the poly(*N*‐acryloyl glycinamide)‐based hydrogel with initial monomer concentration of 40% mol and different NAGA/THMMA mass ratios. Reproduced with permission.^[^ ^188^ ^]^Copyright 2018, John Wiley and Sons. C) Macroscopic appearance and mechanical performance of the printed porous poly(I‐acryloyl glycinamide)‐based hydrogel scaffolds (scale bar = 2 mm). The 3D printed scaffolds show excellent mechanical properties: C1,C2) twisting and D1,D2) compression. D) The materials are biocompatible; here it shows cell adhesion and proliferation of human bone marrow stem cells (hBMSCs) cultured on hydrogel scaffold (scale bar = 200 µm).

Liu and co‐workers reported on a newly designed thermoresponsive *N*‐acryloyl glycinamide‐based supramolecular copolymer hydrogel (**Figure** **7**A).^[^ ^188^ ^]^ As their previous study suggested, poly(*N*‐acryloyl glycinamide) (PNAGA) has a high softening temperature and was difficult to process via the extrusion method,^[^ ^189^ ^]^ *N*‐[tris(hydroxymethyl)methyl] acrylamide (THMMA) was thus copolymerized with NAGA. The hydrogen bonds from the PNAGA network could be broken upon heating, while it could also rapidly recover upon cooling, resulting in a reversible gel‐to‐sol transition. Due to the presence of multiple hydrogen bonds, the obtained copolymer hydrogels demonstrate high tensile strength (up to 0.41 MPa), large stretchability (up to 860%), and high compressive strength (Figure 7B,C). Growth factor beta 1 (TGF‐*β*1) and *β*‐tricalciumphosphate (*β*‐TCP) were incorporated into the ink, which stimulated the proliferation and differentiation of human bone marrow stem cells (hBMSCs) (Figure 7D). The hydrogel scaffolds could promote the regeneration of both cartilage and subchondral bone within osteochondral defects (Figure 7D). Using a similar method, the same authors printed ink composed of NAGA and nanoclay.^[^ ^190^ ^]^ The hydrogen bonding combined with the physical crosslinking of nanoclay contributed to the superior mechanical performances of 3D printed hydrogel with tensile strength, Young's modulus, and break strain of up to 1.17, 0.18 MPa, and 1300%, respectively. Not only could the 3D printed devices promote the osteogenic differentiation of primary rat osteoblast (ROB) cells, but it could also facilitate the regeneration of new bone in tibia defects of rats.

#### 3.3.2 Mechanical Properties and Conductivity

Mechanical properties are crucial for electrical and conductive polymers, especially if they are to be developed as soft electronic devices. Kong and co‐workers developed a tough conductive hydrogel based on polyacrylamide (PAA)‐alginate for 3D printing.^[^ ^191^ ^]^ A microfluidic device was used as the 3D printer head, which enables the fabrication of hydrogel microfibers and other complex 3D microstructures. The printed hydrogel microfibers show extremely high stretchability (2100%), which could be ascribed to the dissipation of mechanical energy by reversible Ca^2+^ crosslinking and maintaining of the elasticity from the covalently crosslinked PAA network. The toughness of the hydrogel was also estimated as 1800 kJ m^−3^. An interesting experiment showed a 60 g egg dropped from a height of 1 m onto the printed web bouncing several times on the tough hydrogel web and remaining intact. The hydrogels can be conductive after an electrolyte treatment, such as a KCl aqueous solution. These hydrogels can be used to fabricate sensors that detect movement of a human elbow. Advincula and co‐workers utilized DIW to process tough, elastic and conductive polyurethane foam.^[^ ^133^ ^]^ The printable ink was formulated by dispersing nanoclay and silica nanoparticles in a concentrated solution of polyurethane. The printed polyurethane foam shows unprecedented elasticity (1000 cycles, 60% compression strain) and robustness (compressive modulus of 60 KPa), which could be attributed to a unique hierarchical porous morphology generated by acid etching and a phase inversion process. After compositing the material with CNTs, conductivity was induced in the elastic 3D printed devices showing a change of electrical resistance as a function of compressive stress. It is worth noting that such a printed material system is much more sensitive than previously reported highly sensitive graphene/polymer sponges.^[^ ^192^ ^]^ Even though the electrical conductivity of the 3D printed electronic devices is typically caused by graphene doping, it is still highly desirable for alternative 2D nanomaterial systems to be considered. For example, hexagonal boron nitride (hBN) is lightweight, abundant, readily commercially available, and has a high thermal conductivity of up to 2000 W (m K)^−1^. Hu and co‐workers demonstrated the use of 3D extrusion printing technology to fabricate wearable textiles based on boron nitride (BN)/PVA composites.^[^ ^193^ ^]^ Transmission electron microscopy (TEM) and small‐angle X‐ray scattering (SAXS) results suggest good orientation of BN nanosheets in 3D printed textiles after uniaxial elongational flow during fiber printing and further hot‐drawing processing. The highly aligned boron nitride and dense 3D printed fiber structures not only significantly improve the mechanical properties, with a measured tensile strength of 355 MPa (significantly higher than that of pure PVA), but also facilitated heat transfer along the fibers with a 55% higher cooling effect than that of a commercial cotton fabric.

#### 3.3.3 Mechanical Properties, Conductivity, and Biocompatibility

To be developed as effective implantable bioelectronic devices, it is advantageous that the polymer materials have combined advanced properties, including desirable mechanical properties, conductivity, and biocompatibility. Conductivity is especially required when the biocompatible, tough 3D hydrogel scaffolds are used to support the growth of electro‐responsive cells such as nerve and muscle cells.^[^ ^194^ ^]^ Therefore, graphene, a single layer 2D nanomaterial with extraordinary electrical conductivity, mechanical strength, as well as the ease of functionalization, has been incorporated into polymer matrices. Wallace and co‐workers studied the effect of graphene addition on the properties of 3D printed poly(trimethylene carbonate) (PTMC) based composites.^[^ ^195^ ^]^ It was found that the addition of graphene not only resulted in significantly enhanced tensile strength and electrical conductivity (≈1 × 10^−1^ S m^−1^ with 3 wt% graphene) without altering the 3D printability, but also promoted the mesenchymal stem cell (MSC) attachment and proliferation. In other work, the authors further demonstrated that the mechanical (tensile strength increased from 47.8 to 75.7 MPa with addition of 3 wt% graphene), electrical properties (conductivity increased from 1 × 10^−8^ to 2.5 × 10^−1^ S m^−1^ with addition of 3 wt% graphene), as well as the adhesion, proliferation and spreading of L929 fibroblasts cells of chitosan hydrogels composites could be improved through graphene addition.^[^ ^196^ ^]^

Shah and co‐workers reported 3D printing of mechanically robust, conductive and biocompatible graphene composites, using an ink consisting of a hyper‐elastic elastomer, polylactide‐*co*‐glycolide (PLG), and graphene dissolved in dichloromethane (DCM).^[^ ^197^ ^]^ Rapid evaporation of DCM following the extrusion is critical to creating self‐supporting fibers that do not significantly deform following layer‐by‐layer deposition. These showed excellent flexible elastic properties (elastic modulus of 16 MPa) and both in vitro (supporting the growth and differentiation of human mesenchymal stem cells) and in vivo biocompatibility (no immune response). The printed composite exhibits electrical conductivities greater than 800 S m^−1^, which is still maintained after several cycles of mechanical deformations. The same authors mixed PLG with hexagonal boron nitride. This was then 3D printed through the extrusion method to obtain various complex structures.^[^ ^198^ ^]^ The printed composites were demonstrated to have multiple advanced properties including high flexibility and stretchability, thermal conductivity (up to 2.1 W K^−1^ m^−1^), and good cytocompatibility.

#### 3.3.4 Self‐Healing Combined with Other Advanced Properties

**Figure 8:**
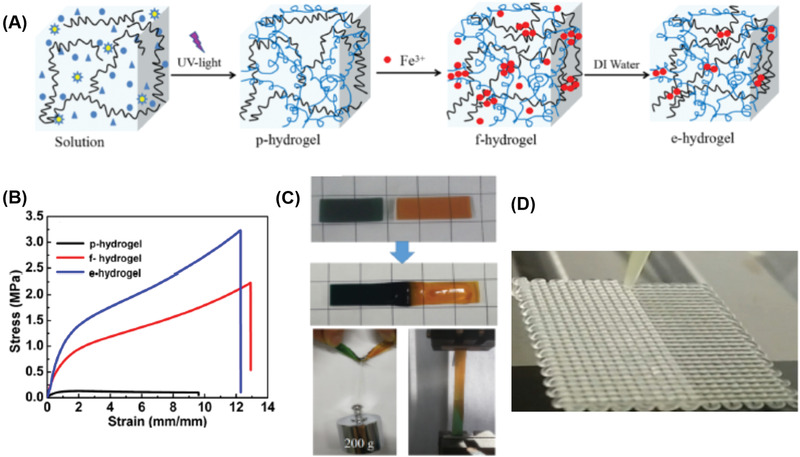
3D printed double crosslinked alginate inks with tunable mechanical and self‐healing properties via the crosslink density and interactions. A) Synthesis scheme of the metal coordination interaction in a dual ion crosslinked sodium alginate/poly(acrylamide‐*co*‐acrylic acid)/Fe^3+^ hydrogel. Reproduced with permission.^[^ ^201^ ^]^ Copyright 2019, American Chemical Society. B) Typical tensile stress−strain profiles of sodium alginate/poly(acrylamide‐*co*‐acrylic acid)/Fe^3+^ hydrogel. C) Healing properties of sodium alginate/poly(acrylamide‐*co*‐acrylic acid)/Fe^3+^ hydrogel. The photos show the healing process at room temperature for 48 h. D) Photo in the 3D printing process.

Self‐healing materials are a class of smart materials that can recover to their original functionality after breakage. Developing versatile 3D printing materials with intrinsic self‐healing properties is a promising strategy to expand their applications. Liu and co‐workers reported the 3D printing of poly (*N*‐acryloyl glycinamide‐*co*‐2‐acrylamide‐2‐methylpropanesulfonic) (PNAGA‐PAMPS) hydrogels, which exhibit self‐healing, conductivity, toughness and cytotoxic properties.^[^ ^199^ ^]^ The strong hydrogen bonding between the NAGA and AMPS units results in reversible sol–gel transitions and self‐healing. While doping with poly(3,4‐ethylenedioxythiophene)‐poly‐(styrenesulfonate) (PEDOT‐PSS) resulted in electrical conductivity. Constructing the double physically crosslinked network is another attractive strategy to impart multifunctionality onto hydrogels. Wu and co‐workers prepared a novel double crosslinked hydrogel sodium alginate/poly(acrylamide‐*co*‐acrylic acid) system, utilizing the metal coordination between Fe^3+^ and both alginate and acrylic acid (**Figure** **8**A).^[^ ^200^ ^]^ By optimizing the chemical composition of hydrogels, as well as the Fe^3+^ concentrations, outstanding mechanical properties were achieved with good tensile strength (3.24 MPa) and strain (1228%) (Figure 8B). Furthermore, the reversible, Fe^3+^−COO^−^ ionic crosslinker not only enables the hydrogels to be self‐healable, indicated by the joining of two separated pieces into one (Figure 8C), but also allows for a 64% recovery in toughness after 4 h. The hydrogels were successfully 3D‐printed into various architectures through adjusting viscosity and gelation rates (Figure 8D).

Excellent elasticity and self‐healing properties were demonstrated in 3D printable silica/poly(tetrahydrofuran)/poly(e‐caprolactone) (SiO2/PTHF/PCL‐diCOOH) hybrid materials.^[^ ^201^ ^]^ The hybrids were prepared by sol–gel and an in situ cationic ring opening polymerization (CROP). The specific combination of various noncovalent bonding interactions (London dispersion forces, dipole–dipole interactions and hydrogen bonding) between the polymer chains enables the hybrid materials to have excellent self‐healing properties. In addition, the combination of these noncovalent interactions and the strong covalent bonds between the SiO_2_ inorganic and polymeric chains, superior elasticity was demonstrated, which showed elastomeric deformation under tension and the ability to recover the initial shape when the load was released. Besides excellent mechanical properties and self‐healing, the biocompatibility of 3D printable polymers could be achieved through constructing the double network consisting of host–guest interaction and covalent bonds.^[^ ^105^ ^]^

**Figure 9:**
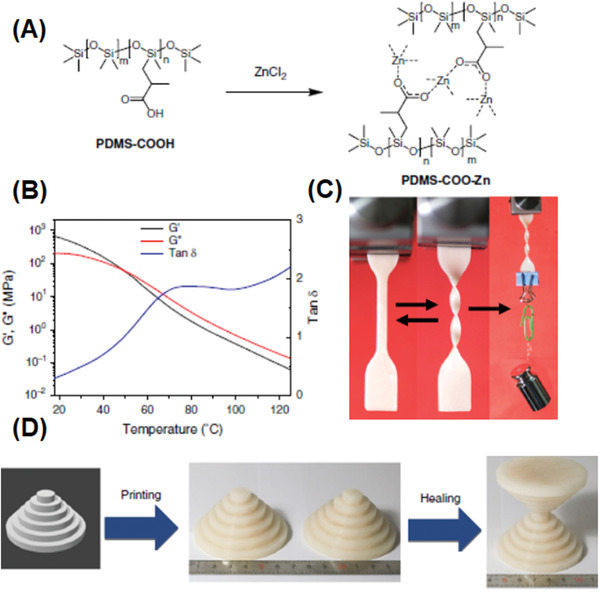
The use of Zn(II)‐carboxylate interactions to impart multifunctionalities into 3D printed polymers. A) Synthesis and structure of PDMS polymers crosslinked using Zn(II)‐carboxylate interactions. Reproduced with permission.^[^ ^202^ ^]^ Copyright 2018, Nature Publishing Group. B) Temperature dependent rheology measurements of PDMS–COO–Zn polymer. C) A fusilli‐shaped polymer made by local heating within tens of seconds can sustain a weight of 100 g immediately. D) The irregular objects obtained by 3D printing.

Utilizing noncovalent bonds (electrostatic interaction, hydrophobic interaction, and hydrogen bonding) represents another innovative method to control material properties in 3D printable polymers. Zuo and co‐workers designed a PDMS polymer by using Zn(II)‐carboxylate interactions as the crosslinker (**Figure** **9**A).^[^ ^202^ ^]^ These weak but abundant Zn(II)‐carboxylate interactions endow the polymer with high mechanical strength (Young's modulus of 478.13 ± 20.25 MPa and a storage modulus of 470 MPa at 25 °C, Figure 9B,C). When heated, the coordination equilibrium shifts towards the disassociated state, increasingly generating non‐crosslinked PDMS‐COO^−^ chains and reducing the mechanical strength (0.06 MPa at 125 °C). Such a significant mechanical property change enables the material to heal itself when damaged upon heating. When the sample is healed at 80 °C for 4 h, the breaking strain and maximal strength could be completely recovered to the original values. This thermal sensitive Zn(II)‐carboxylate coordination equilibrium also facilitates the processability by FFF. Various irregular shapes could be printed, which also can be thermally annealed when damaged (Figure 9D). The electrical conductivity could be induced through adding graphene, which increased the electrical conductivity to as high as 850 S cm^−1^. Lei and Wu have also developed a novel type of zwitterionic hydrogel, composed of methacrylic acid (MAA) and 3‐dimethyl (methacryloyloxyethyl) ammonium propanesulfonate (DMAPS) units.^[^ ^40^ ^]^ When the content of MAA is 40% mol, the hydrogel is mainly crosslinked by ionic interactions and hydrophobic associations, which exhibited ultrahigh stretchability (>10 000% strain) and relatively high strength (>0.1 MPa). Rheology results confirmed the shear‐thinning behavior at 60 °C. The high viscosity (≈2000 Pa s) was observed at a low shear rate (<0.1 s^−1^), whereas at a high shear rate (≈300 s^−1^), the viscosity decreases to ≈14 Pa s. These results indicated that the hydrogel is suitable for DIW and shape retention after extrusion. Self‐healing at room temperature, within 12 h, was observed. Various integrated properties, including stimuli‐responsiveness, conductivity, biocompatibility, and antibacterial activity were also demonstrated. Incorporating ion–dipole interactions and ionic liquid into the fluorocarbon elastomer results in a number of functionalities including intrinsically conductive, transparent, stretchable, and self‐healing capabilities in aquatic conditions.^[^ ^203^ ^]^ Printed circuit boards made using poly(vinylidene fluoride‐*co*‐hexafluoropropylene) and ionic liquid inks, could light up LEDs under water even after sustaining damage.

### 3.4 Dynamically Tunable Properties

Conventional 3D‐printed objects retain the same properties and structures during their entire product lifetimes. Recently, attention has increasingly been paid to developing the capability to modulate the properties of 3D printed devices in a temporally dependent manner. To achieve this, stimulus‐responsive polymers which could change their properties and structure in response to external stimuli have emerged as an intriguing class of materials for 3D printing. Combining the stimuli‐responsive properties with advanced 3D printing techniques not only significantly expands the range of applications for conventional smart polymers, but also holds great promise in meeting the ever‐increasing additive manufacturing demand of complex device platforms. Interested readers can refer to the review about 3D printing of stimulus‐responsive polymers.^[^ ^204^ ^]^ In this section, we introduce and discuss the achievement of novel dynamic properties of 3D printed objects.

#### 3.4.1 Shape Transformation

Polymer actuators have a unique capability to change shape or undergo motion in response to external stimuli. These represent one of the most important classes of smart materials.^[^ ^205^, ^206^ ^]^ Such materials have been demonstrated to mimic the movement of a diverse range of living organisms, with promise for a wide range of potential applications in soft robotics,^[^ ^206^ ^]^ artificial muscles,^[^ ^207^ ^]^ 3D cell culture,^[^ ^187^, ^208^ ^]^ and drug or cell delivery devices.^[^ ^209^ ^]^ 3D printing of shape changing polymers, also termed 4D printing,^[^ ^16^, ^210^, ^211^, ^212^, ^213^, ^214^, ^215^, ^216^, ^217^ ^]^ is an emerging technology with a great capacity for fabricating complex 3D structures, providing great potential for a wide range of applications. Various shape changing polymers including hydrogels,^[^ ^98^, ^99^, ^111^, ^117^, ^118^, ^188^, ^189^, ^190^, ^191^, ^192^, ^193^, ^194^, ^195^, ^196^, ^197^, ^198^, ^199^, ^200^, ^201^, ^203^, ^204^, ^205^, ^206^, ^207^, ^208^, ^209^, ^210^, ^211^, ^212^, ^213^, ^214^, ^215^, ^216^, ^217^, ^218^, ^219^, ^220^ ^]^ shape memory polymers,^[^ ^153^, ^221^, ^222^, ^223^, ^224^, ^225^, ^226^, ^227^ ^]^ liquid crystal elastomers,^[^ ^228^, ^229^, ^230^, ^231^, ^232^, ^233^ ^]^ and silicone‐based elastomers^[^ ^234^, ^235^, ^236^, ^237^ ^]^ have been demonstrated to undergo 4D printing.

##### Printing of Dynamic Structures

**Figure 10:**
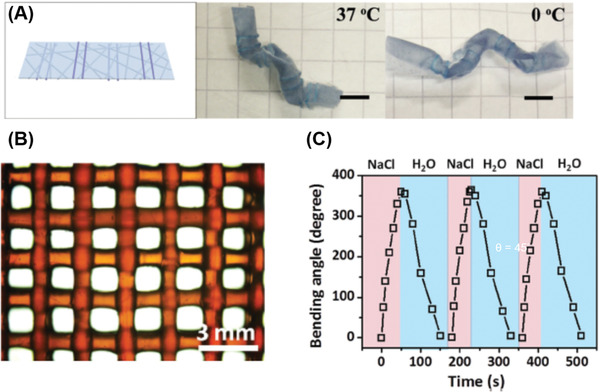
Introducing stresses into a printed 2D structure using extrusion‐based 3D printing. A) Irreversible formation of folded tube with sites for thermo‐actuations by printing poly(*N*‐isopropyl acrylamide) (PNIPAm) hydrogel lines on both sides of the electrospun substrate (scale bars: 5 mm). Reproduced with permission.^[^ ^218^ ^]^ Copyright 2016, John Wiley and Sons. B) Micrograph of the two‐layer printed grid of poly(acrylic acid‐*co*‐acrylamide) hydrogel fabricated by 3D printing.^[^ ^111^ ^]^ C) Variation of bending angles of the printed poly(acrylic acid‐*co*‐acrylamide) hydrogel constructs with time after cyclic incubation in 4 m saline solution and pure water.

One type of 4D printing relies on introducing nonuniform stresses into a printed 2D structure to trigger the formation of 3D structures. Compared to traditional sophisticated fabrication technologies, such as photolithography,^[^ ^238^ ^]^ ion printing,^[^ ^239^ ^]^ and Lego‐inspired assembly,^[^ ^240^ ^]^ 3D printing offers excellent flexibility in designing complex patterns. With the aid of 3D extrusion printing, the inhomogeneous structure could be easily generated and programmed in the horizontal direction of the polymer films in a single step. Agarwal and co‐workers reported the 3D printing of diverse elaborate patterns on the surface of poly(*N*‐isopropyl acrylamide) electrospun membranes.^[^ ^218^ ^]^ The printed PNIPAm polymers have a lower swelling ratio than that of the underlying PNIPAm‐based membrane because of the higher physical crosslinking density. As a result, the swelling/shrinkage mismatch between the printed and underlying layer would be generated, causing thermally induced shape deformation of the printed bilayer hydrogels. Taking advantage of the outstanding designability by 3D printing, parallel lines of PNIPAm polymers with different swelling ratio were printed on both sides of the PNIPAm‐based membrane (**Figure** **10**A), resulting in complex 3D zigzag structures upon heating.

3D printing could also enable the simultaneous deposition of multiple materials, which is very difficult to be achieved by traditional methods. Huang and co‐workers printed 2D patterns consisting of poly(*N*‐isopropylacrylamide) (pNIPAAm)–Laponite and pNIPAm–Laponite−GO nanocomposite hydrogels.^[^ ^117^ ^]^ Due to the swelling ratio difference, circular patterns with alternating annular filaments could be transformed into a complex 3D hyperbolic paraboloid structure upon heating. Multiple liquid crystal polymeric inks with different phase transition temperatures have also been incorporated into a single printed structure.^[^ ^241^ ^]^ The sequential, reversible and multiple shape changes were induced upon heating. Using extrusion‐based 3D printing, other shape memory polymers such as PLA nanocomposites,^[^ ^224^, ^226^ ^]^ polymer blends,^[^ ^225^ ^]^ and epoxy composites^[^ ^153^ ^]^ have been extruded and printed into complex 2D patterns, generating various programmable 3D shapes upon various stimuli. Wu and co‐workers fabricated layer‐by‐layer grid salt‐responsive hydrogel patterns DIW (Figure 10B).^[^ ^111^ ^]^ Poly(acrylic acid‐*co*‐acrylamide) and poly(acrylic acid‐*co*‐*N*‐isopropyl acrylamide) solutions were used as the ink, which were immediately incubated in an FeCl_3_ solution to trigger ionic gelation. The physically crosslinked hydrogels not only have excellent mechanical performance (tensile breaking stress = 2.38 MPa, breaking strain = 802%, and elastic modulus = 0.80 MPa), but also strong interfacial bonding. These also undergo rapid shape change, only requiring tens of seconds for the printed hydrogels to undergo 3D deformation (Figure 10C), which is much faster than most reported hydrogels actuators. The fast response speed could be ascribed to the fabrication of high‐resolution micrometer‐sized gel fibers (Figure 10B).

Generating gradient heterogeneous structures in the vertical direction is another efficient strategy to induce 3D deformation of printed 2D structures.^[^ ^242^, ^243^ ^]^ Leonid and co‐workers reported the 3D printing of alginate and hyaluronic acid hydrogels, which are one of the most frequently used biopolymers.^[^ ^187^ ^]^ Both the alginate and hyaluronic acid polymers were functionalized with methacrylate groups to enable subsequent photo‐crosslinking. The 3D printability was assessed by rheological measurements, which shows that the polymer solution viscosity decreases at increased temperatures and with decreased polymer concentrations. After the polymers were extruded from the nozzle and printed onto a substrate, photo‐crosslinking was initiated using green light. Self‐folding of the crosslinked polymer films was observed in seconds after immersion in water, which could be ascribed to the formation of a crosslinking gradient across the film thickness during the light irradiation process. The unique advantage of using 3D extrusion printing was demonstrated with the ability to fabricate many self‐folded hollow tubes on the glass substrate. Such self‐folded tubes, with inner diameters ranging from 20 to 150 µm, were demonstrated to support cell survival for at least 7 days without any decrease in cell viability. They also showed that incorporating sodium chloride particles into ink formulations could result in the formation of gradient porous structures after soaking in DI water, which are responsible for the rapid, programmable bending movement of 3D printed objects.^[^ ^70^ ^]^

**Figure 11:**
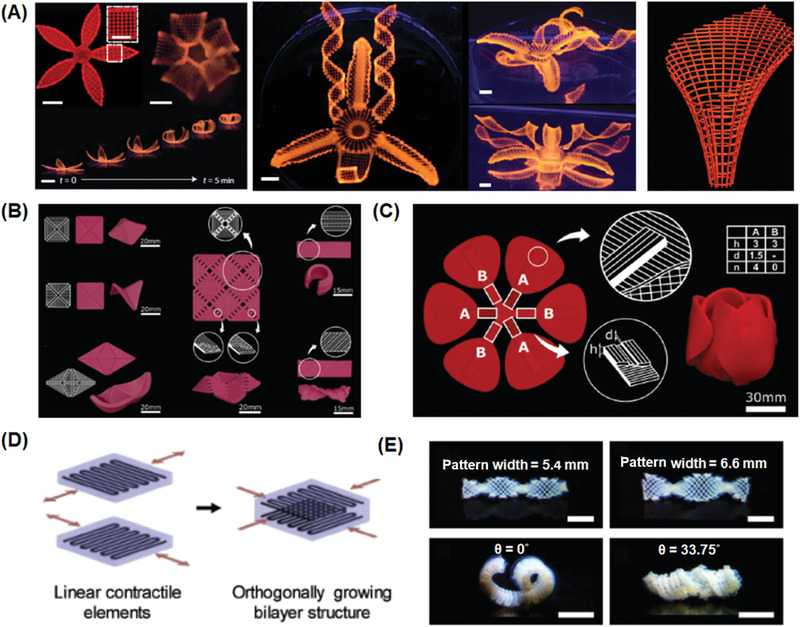
Complex shape deformations of printed 2D structures. A) Complex 3D morphologies generated by biomimetic 4D printing of cellulose poly(*N*‐isopropylacrylamide) hydrogels (scale bars = 5 mm). Reproduced with permission.^[^ ^244^ ^]^ Copyright 2016, Nature Publishing Group. B,C) Complex DNA‐inspired shapes through controlling the complex patterns and orientation of PLA filaments toward the semipassive panels (scale bars = 20 mm in (B)). Reproduced with permission.^[^ ^227^ ^]^ Copyright 2018, Royal Chemical Society. D) Schematic illustrating the 3D printing process to create 3D structures using a bilayer structure with different orientations between each layer. Reproduced with permission.^[^ ^245^ ^]^ Copyright 2019, John Wiley and Sons. E) Various 3D complex shapes could be achieved through controlling the width and angle between the long axis of a bilayer structure in (D) and the direction of the intrinsic curvature (scale bars = 5 mm).

**Figure 12:**
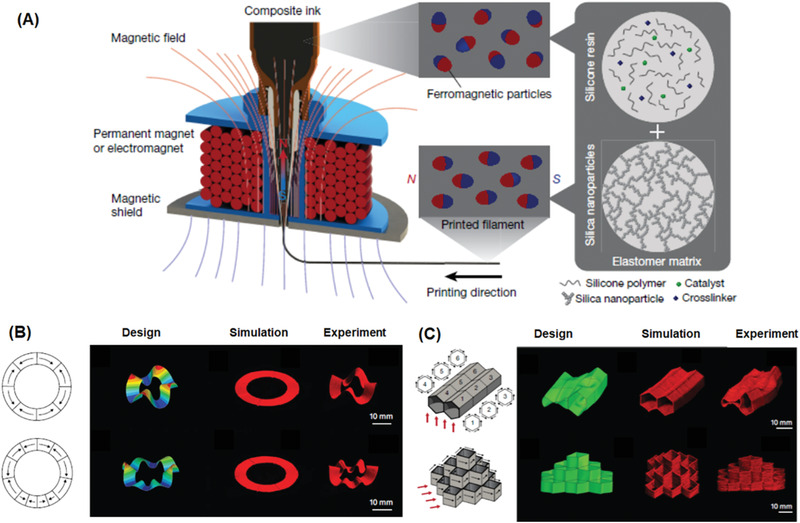
3D printing of elastomer composites containing ferromagnetic microparticles. A) Schematics of the printing process and the material composition. The ferromagnetic particles embedded in the composite ink are reoriented by the applied magnetic field generated by a permanent magnet or an electromagnet placed around the dispensing nozzle. Reproduced with permission.^[^ ^236^ ^]^ Copyright 2019, Nature Publishing Group. B) Schematic designs, finite‐element simulations and experimental results for an annulus encoded with two annular rings with the same geometry but different patterns of ferromagnetic domains. C) Schematic designs, finite‐element simulations, and experimental results for two adjoining hexagonal tubes programmed to form undulating surfaces under the applied magnetic field owing to the alternating ferromagnetic domains; and a pyramid‐shaped thin‐walled structure exhibiting elongation in its diagonal direction along the applied magnetic field).

Because the size, shape, and organization of printed features could be easily designed through computer aided 3D extrusion printing, more complex 3D actuated movement can be achieved. Lewis and co‐workers printed a hydrogel composite consisting of *N*,*N*‐dimethylacrylamide and nanofibrillated cellulose.^[^ ^244^ ^]^ The printed bilayer structure consisting of anisotropic gel fibers generates a 3D curvature structure due to the differential swelling between the top and bottom layers. Such anisotropic hydrogel fibers could be easily printed into diverse complex 2D patterns including concentric circles, saddle‐like shapes, complex flowers and other biomimetic morphologies (**Figure** **11**A). The excellent versatility of 4D printing was demonstrated as the ability to mimic the sophisticated structure of both the orchid Dendrobium helix and calla lily flower. Using the FFF method, shape memory PLA could be extruded and printed on the surface of a semipassive bottom layer through a cheap and simple hobbyist printer.^[^ ^227^ ^]^ By controlling the complex patterns and orientation of PLA filaments toward the semipassive panels, various pre‐programmable 3D shapes have been produced including self‐folding origami, DNA‐inspired shapes, pyramids, saddle shapes, boats, and sequential folding (Figure 11B,C).^[^ ^227^ ^]^ The complex bioinspired shape motions could also be realized by combining two printed layers with different orientations (Figure 11D).^[^ ^245^ ^]^ A wide range of sophisticated motions from simple bending to coiling and twisting were achieved (Figure 11E). Zhao and co‐workers developed DIW of an elastomer composite containing ferromagnetic microparticles to enable shape changing.^[^ ^236^ ^]^ The magnetic particles could be reoriented by applying a magnetic field to the dispensing nozzle, enabling the alignment of ferromagnetic domains in complex magnetization patterns (**Figure** **12**A). A simulation model was developed to predict the transformation of complex 3D‐printed structures with programmed ferromagnetic domains under magnetic fields. It was shown that the magnitude of annular rings could affect the macroscale actuation behavior despite being of the same geometry. When programmed into more intricate domain patterns (Figure 12B), complex shape changes were induced under applied magnetic fields such as miura‐ori, out‐of‐plane morphing hollow crosses, quadrupedal and hexapedal structures.

The orientation of liquid crystalline elastomers (LCE) can be aligned along the direction of the print path due to shear forces during the DIW process, enabling programmable control over their complex actuation behaviors. Lewis and co‐workers demonstrated the spatially programmed nematic order using DIW at a high operating temperature.^[^ ^230^ ^]^ It was shown that the processing temperature affects 3D printability as the LCE ink is a Newtonian fluid when it is above its isotropic transition temperature. The achieved spatial control over the director alignment was confirmed by polarized optical microscopy, which showed the change of transmittance when orienting the crosspolarizers. In addition to this, the programming of complex 3D shape changes was achieved, including a cone with positive and negative Gaussian curvature and out‐of‐plane morphing conical arrays. Using a similar method, Sánchez‐Somolinos and co‐workers printed thermally responsive main‐chain LCEs on top of a nonresponsive commercial polymer film.^[^ ^231^ ^]^ The printed LCE undergoes a thermal contraction along the filament printing direction when heated above its isotropic transition temperature (TN1). Diverse complex director patterns varying in‐plane has been successfully fabricated by 3D printing, leading to sophisticated shape‐morphing functions such as cone membranes, rotation of the central annular object and bulging out of the PDMS toward the side far from LCE patterns. Hagaman et al. reported, a photoresponsive azobenzene‐containing LCE could be printed on the surface of a Kapton film.^[^ ^229^ ^]^ Due to the fully reversible trans–cis photoisomerization of azobenzene upon irradiation of light with different wavelength,^[^ ^246^ ^]^ the bilayer structures exhibits rapid actuation with full cycles completed within seconds. It was found that a lengthy alkyl spacer between the AB mesogen affects the photo‐induced bending angle and photo generated stresses.^[^ ^228^ ^]^ Qi and co‐workers showed actuation using a liquid crystal elastomer 3D printed onto a rotating mandrel, creating a long fiber.^[^ ^247^ ^]^ It was demonstrated that the fibers exhibited reversible actuation and excellent mechanical properties. Furthermore, the fibers were woven to form a variety of smart textiles that can help regulate increased body temperatures. For these aforementioned examples, continuously heating above nematic to isotropic transition temperature is required to keep the 3D actuated shapes, which has an associated energy cost. Most recently, Lewis and co‐workers proposed a novel approach of using dynamic exchangeable bonds to address this issue.^[^ ^248^ ^]^ They used an allyl disulfide group, which could undergo associative bond exchange upon UV irradiation, allowing network reconfiguration and fixing the thermally actuated 3D shapes of liquid crystal elastomers.

#### 3.4.2 3D Printing Dynamic Structures

One of the key advantages of 3D printing over other traditional microfabrication technology is the ability to fabricate 3D objects. 3D printing shape changing polymers enables further 3D shapes to be programmed under external stimuli, allowing complex geometries that otherwise would normally be difficult to achieve. By stacking up layers of deposited filaments, Zhao and co‐workers printed the ferromagnetic domains into 3D multilayered geometries (Figure 12C).^[^ ^236^ ^]^ To enhance the dimensional stability of supporting filaments, the fumed silica nanoparticles were introduced into a support ink. When the two high aspect ratio hexagonal tubes with different actuation behavior were assembled, complex undulating surfaces were generated under the applied magnetic field (Figure 12C). The versatility of the 3D extrusion printing method also enables fabricating auxetic structures with negative Poisson's ratios, which is very difficult to achieve by other fabrication techniques. The printed auxetic structures exhibit very fast shrinkage in both length and width in response to external magnetic fields (0.5 s). Ware and co‐workers fabricated positive and negative Gaussian curvatures of LCE through DIW printing.^[^ ^228^ ^]^ A hollow LCE 3D hemisphere with a +1 defect pattern was morphed into a “peaked” hemisphere when heated above TN1, resulting from deformation of the sample and retention of the existing positive Gaussian curvature. The 3D geometry which contains a region of positive Gaussian curvature connected to a region of negative Gaussian curvature, was designed and printed. Notably, the LCE with such unique structures exhibit repetitive snap‐through shape transition that are not observed in traditionally fabricated LCEs.

**Figure 13:**
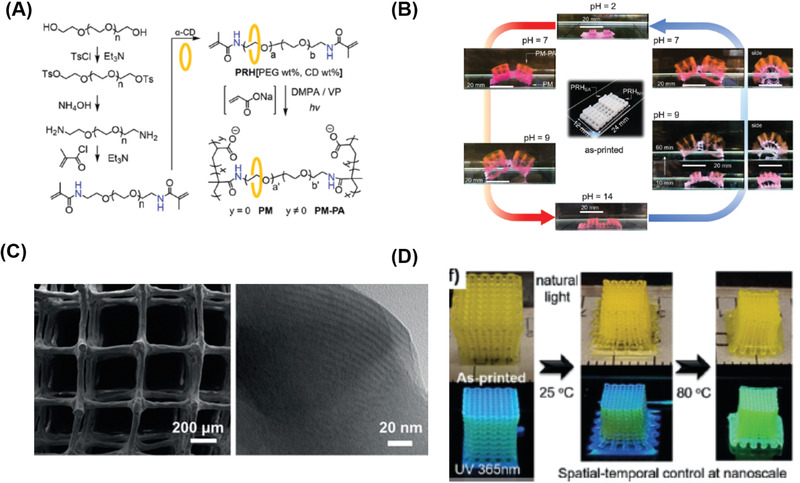
The shape changing of printed 3D supramolecular hydrogel structures. A) Synthesis of pH responsive PEG‐(NH‐MA)2, *α*‐CD threaded polypseudorotaxane hydrogels. Reproduced with permission.^[^ ^99^ ^]^ Copyright 2017, Royal Chemical Society. B) Images of a designed hybrid hydrogel in its as‐printed form and crosslinked forms at different pH. C) 3D printed hierarchical micro and nano structures (left, SEM; right, TEM). Reproduced with permission.^[^ ^97^ ^]^ Copyright 2018, John Wiley and Sons. D) A 3D printed wood pile lattice cube underwent volume reduction and a change in the fluorescence color upon evaporation.

Supramolecular interactions such as ionic interactions, hydrogen bonding, guest/host interactions, and *π*−*π* stacking have recently received a growing amount of attention in the field of polymer chemistry.^[^ ^249^, ^250^ ^]^ Not only does the dynamic nature of noncovalent interactions facilitate the additive manufacturing of 3D objects due to advantageous rheological performance, but also novel material functionalities could also be integrated, such as self‐healing, biocompatibility, toughness and macroscopic actuation. Ke and co‐workers designed and synthesized a novel type of polypseudorotaxane hydrogels, based on supramolecular inter‐ring hydrogen‐bonding interactions between *α*‐cyclodextrins and poly(ethylene oxide) units.^[^ ^98^ ^]^ The rheological properties of the ink consisting of Pluronic F127 (poly(ethylene oxide) (PEO)–poly(propylene oxide) (PPO)–PEO) and *α*‐cyclodextrins were studied. It was shown that through controlling concentrations of F127 and *α*‐cyclodextrins, the printability could be significantly improved, which was confirmed by its shear‐thinning properties and fast self‐recovery. After soaking the printed cubic woodpile lattices in dimethyl sulfoxide (DMSO) for 24 h, the 3D structures were deformed into transparent hydrogels caused by the disassembly of tubular *α*‐CD polyrotaxane. Interestingly, adding water again into the system generates a very strong recovery force, allowing much heavier objects to be lifted. Furthermore, since the polyrotaxane were formed by interactions of PEO and *α*‐cyclodextrins, it is also sensitive to pH changes due to the deprotonation of the hydroxyl groups in *α*‐cyclodextrins. The same authors demonstrated the rapid and reversible contraction–expansion shape morphing of 3D‐printed polyrotaxane upon pH variation (**Figure** **13**A).^[^ ^99^ ^]^ The incorporation of acrylate moieties into the 3D printing hydrogel enables the shape of printed objects to be further controlled by the ionic strength (Figure 13B). Supramolecular inks incorporating tetraethyl orthosilicate (TEOS) into Pluronic F127 have also been demonstrated^[^ ^97^ ^]^ (Figure 13C). The co‐assembly process between TEOS and Pluronic F127 resulted in the formation of hydrogels, which were subsequently printed into woodpile lattice cubes. Solvent evaporation and calcination after printing initiates further promotes hierarchical co‐assembly, achieving well‐defined high resolution micro–nano structures that are far beyond the physical limit of printing nozzles. The printed objects could undergo simultaneous color‐ and shape‐change upon solvent evaporation due to the presence of fluorescent trackers (Figure 13D).

**Figure 14:**
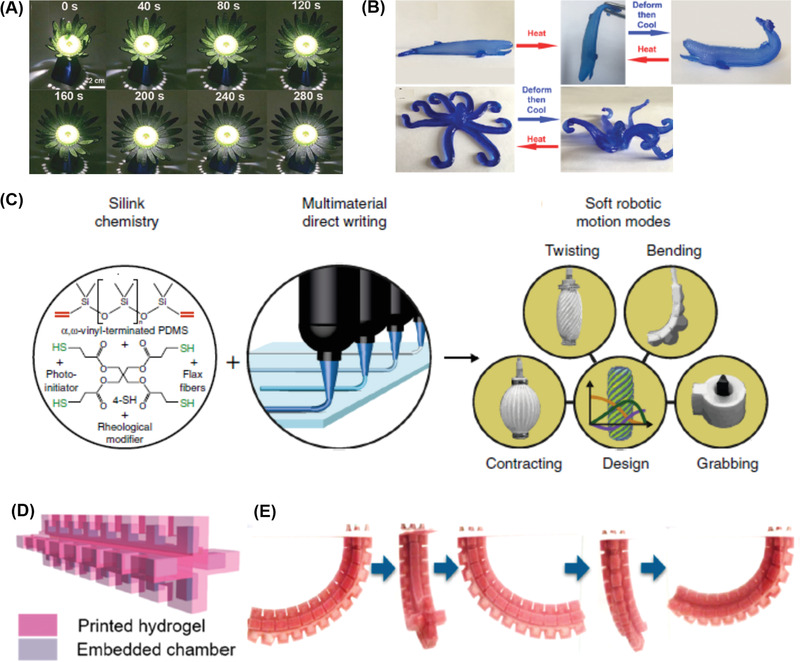
Diverse actuators fabricated by 3D extrusion‐based printing inspired by living organisms. A) Photo‐triggered shape memory behavior of a 3D printed sunflower from the closed to opened state. Reproduced with permission.^[^ ^224^ ^]^ Copyright 2017, John Wiley and Sons. B) Whale and octopus shaped hydrogel was used to demonstrate temperature induced shape change. Reproduced with permission.^[^ ^118^ ^]^ Copyright 2018, American Chemical Society. C) Silicone‐based inks with variable ink constituents yield Silinks with tunable stiffness ranging from soft and stretchable to hard and stiff. Multimaterial 3D printing seamlessly combines materials of different stiffness in a single print, allowing for precise programming of the actuator's shape transformations when inflated. Reproduced with permission.^[^ ^235^ ^]^ Copyright 2018, Nature Publishing Group. D) Schematic diagram of and E) corresponding full circle rotation of the 3D printed artificial tentacle with four‐channel hydraulic connection. Reproduced with permission.^[^ ^251^ ^]^ Copyright 2020, American Chemical Society.

The ability to actively change shape is essential to many living organisms. For example, the Venus flytrap closes its leaves in milliseconds to efficiently catch flies and pinecones to open their scales when the environment is dry to release their seeds. Inspired by such phenomena, various materials and fabrication technologies have been explored to build soft robots, mimicking the actuation functionality of nature's plants. However, it is still very challenging to reach the level of complexity of the programmed architecture found in the systems of nature. 3D printing provides tremendous opportunities for the design and fabrication of biomimetic soft robotics. Chen and co‐workers fabricated 3D sunflower structures with a commercial FFF printer.^[^ ^224^ ^]^ The petals of the sunflower consisted of carbon black‐polyurethane nanocomposites, while the stamen, pedicel and vase of the sunflower were printed with PLA (**Figure** **14**A). The deformation and closing of petals could be induced by application of external force when the temperature of the sunflower was above its *T*_g_, which would then be reversed upon cooling. The shape of the printed sunflower petal would eventually recover to its original state, mimicking hormones of real sunflowers. The movements of the animals could also be mimicked by directly printing the shape‐changing polymers into designed biomimetic structures. Zhang and co‐workers fabricated 3D whale and octopus objects from agarose/polyacrylamide/Laponite inks.^[^ ^118^ ^]^ The dual stimulus thermal/shear‐induced sol−gel transition behaviors of agarose not only resulted in excellent shape stability after printing, but was also able to make the 3D structure to transform its shape into different patterns. The originally closed mouth and straight tailed printed whale‐like hydrogel became open mouthed and cocked its tail after being heated in an oven at 95 °C, while the octopus like hydrogel waved tentacles and seemed to “come alive” (Figure 14B). As a further step forward, Studart and co‐workers direct writing printed the 3D fibrous architectures using a functionalized polysiloxane ink with controlled stiffness (Figure 14C).^[^ ^235^ ^]^ The soft ink formulation was used to generate a highly stretchable cylindrical part, whereas the stiff ink was utilized to create the different programmable fiber architectures on the surface of the soft cylinder at a well‐defined lead angle. Various elaborate shape changing behaviors including elongation, contraction, or twisting motions which mimic complex architectures found in biological systems, were achieved through programming of the lead angle Ho and co‐workers have constructed 3D artificial tentacle with four‐channel hydraulic connections through printing with a rationally designed hydrogel.^[^ ^251^ ^]^ Full circle rotation movement was observed when water flow was controlled through four different channels. Furthermore, 3D robotic heart capable of beating and transporting biomaterials was also fabricated.

#### 3.4.3 Degradation

**Figure 15:**
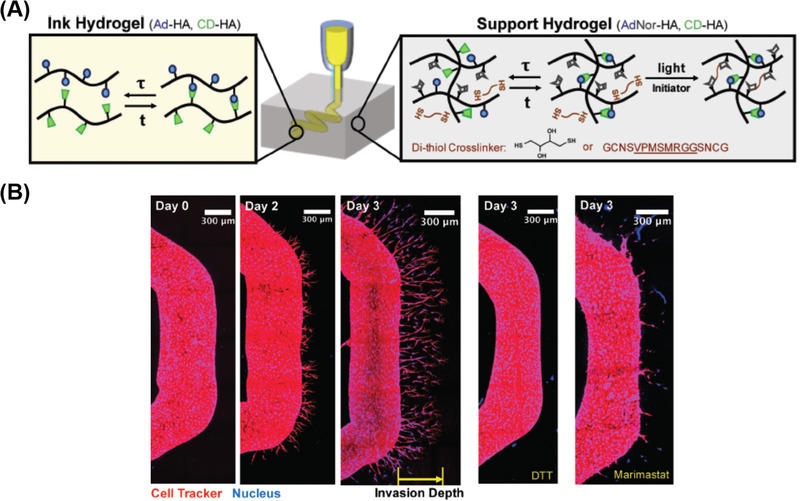
3D printing of biodegradable hydrogels. A) 3D printing process for the deposition of an “ink” hydrogel (yellow) within a “support” hydrogel (gray). Reproduced with permission.^[^ ^256^ ^]^ Copyright 2013, John Wiley and Sons. The ink hydrogel is formulated from the mixing of hyaluronic acid (HA) modified with either adamantane (Ad, blue) (Ad‐HA) or *β*‐cyclodextrin (CD, green) (CD‐HA), whereas the support hydrogel is formulated from the mixing of CD‐HA with HA modified with both Ad and norbornene (Nor, dark gray) groups (AdNor‐HA). B) Representative maximum projection images of EC sprouting from the left channel into the support hydrogel, labeled with cell tracker (red) and Hoechst (nuclei, blue) on days 0, 2, and 3 and with controls of nondegradable crosslinker (DTT) or protease inhibitor (Marimastat) at day 3.

Biodegradability is one of the most crucial and desirable properties in tissue engineering and regenerative medicine that allows the predictable release of encapsulated cells and tethered therapeutics to target injured or diseased tissue through controlled degradation mechanisms.^[^ ^252^, ^253^ ^]^ 3D printing represents a promising rapid prototyping technology to fabricate the scaffolds with complex 3D micro‐nanoarchitectures, which can mimic the extracellular environment. The enzyme cleavable DNA crosslinker was used to prepare physically crosslinked polypeptide hydrogels,^[^ ^113^ ^]^ which not only exhibit excellent 3D printing due to their dynamic nature, but also results in the full on‐demand degradation of the 3D printed hydrogel networks. The collapse of the hydrogel was observed after incubation with a protease which could degrade the polypeptide backbone after twelve hours. Additionally, the nuclease can cleave the DNA linkers and degrade the structure when digested by EcoRI. Furthermore, the release of the encapsulated molecular cargo could be achieved using a novel 3D printed degradable polypeptide–hydrogel construct.^[^ ^254^ ^]^ The hydrogel inks were designed with star architecture to facilitate formation of hydrogels through self‐assembly of the valine domains at the point‐like junctions. The 3D printed hydrogels were degraded when immersed into an isotonic media, with a variety of controlled pH values, due to the deconstruction of polypeptide units and ester bonds. The loaded small molecule drug, doxorubicin hydrochloride (DOX HCl), was found to be released at neutral pH conditions with an initial faster release followed by a steady release. Utilizing the design flexibility of 3D printing, the 2D/3D multiplexed arrays of photodegradable core/shell capsules were fabricated. Due to the efficient photothermal effect caused by the gold nanorods (AuNRs) in the printed core, the programmable release of functional biomolecules was achieved by laser rupture of the underlying poly(lactic‐*co*‐glycolic) acid polymer shell.^[^ ^255^ ^]^ The selective rupture of the capsule and biomolecular releases over an entire array of capsules were demonstrated by controlling the laser irradiation parameters. In order to recapture integrin‐mediated cell adhesion and protease‐sensitive degradation characteristics of ECMs, Burdick and co‐workers fabricated complex microchannels through 3D printing of a newly designed hydrogel ink with both cell adhesion and cell‐mediated degradation abilities (**Figure** **15**A).^[^ ^256^ ^]^ The hydrogels were formed by guest–host interactions between adamantane (Ad) (Ad‐HA) and *β*‐cyclodextrin (*β*‐CD), which was further modified with norbornene to enable the introduction of RGD peptides for adhesion and biodegradable di‐thiol crosslinkers. It was shown that the endothelial cells adhered to the 3D‐printed complex microchannels and formed confluent monolayers. When the cells were exposed to angiogenic factors, they degraded the hydrogel with proteases and formed sprouts into the hydrogel (Figure 15B).

#### 3.4.4 Color Changing

The ability to change color in response to external stimuli is found abundantly in nature and is very important for a wide range of purposes such as intimidation, camouflage, display, and communication. Taking inspiration from nature, scientists strive to use various responsive materials to mimic such functionality. 3D printing enables creating complex structures with multicomponents and multiple color emissions, which are difficult to prepare using traditional manufacturing techniques. Mechanoluminescent polymers containing mechanically sensitive groups, known as mechanophores, are one of the important classes of color changing materials. Boydston and co‐workers printed tensile test specimens which consisted of discrete regions of photo‐ and mechano‐responsive spiropyran groups.^[^ ^257^ ^]^ Mechanical elongation of the printed devices only caused color change in regions containing the mechano‐responsive group, while UV irradiation could activate color change in regions containing photo‐responsive groups. Using a similar strategy, the mechanoluminescent systems composed of PDMS and ZnS particles were printed into 3D devices, with complex structures, in one step.^[^ ^258^ ^]^ Various complex 3D printed geometries exhibited green luminescence under UV exposure at 365 nm and compression. When the multiple materials with different doping levels of ZnS were selectively printed, different patterned color images showed green and orange emissions at designed locations such as a hexagonal mesh and concentric squares. Interestingly, it was shown that such printed hexagonal mesh patterns exhibited anisotropic light emissions upon compression from different directions.

**Figure 16:**
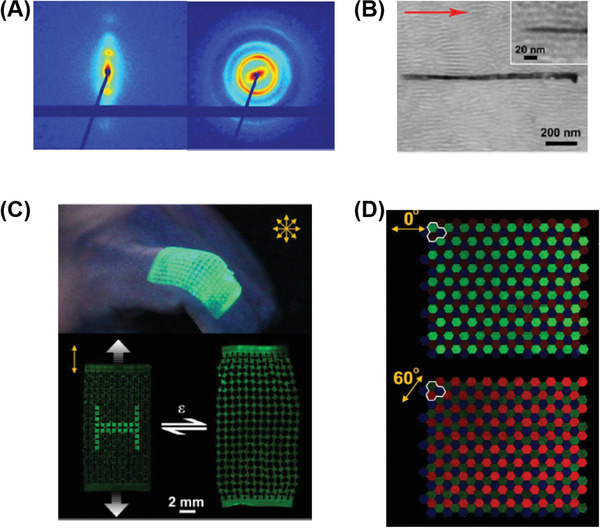
3D printing of color‐changing block copolymer–perovskite crystals composites. Reproduced with permission.^[^ ^260^ ^]^ Copyright 2019, Nature Publishing Group. A) SAXS results of printed pure block copolymer filaments without nanowires. B) TEM image of printed nanocomposite filaments showing perovskite nanowires oriented in parallel with the print direction. C) When viewed using a pair of linear polarizers, the digitally patterned H letter in printed unit cell is displayed (left) or encrypted upon reversible stretching finger motions. D) Optical images of printed pixel arrays showing polarization dependent emission multiplexing upon rotating polarizers.

The nanostructured photonic crystal materials, which have the ability to mimic the structural color observed in nature, were also printed using FFF.^[^ ^259^ ^]^ A novel type of block copolymer ink with self‐assembled nanostructures have been designed and synthesized. The sterically bulky repeat units were incorporated into dendritic copolymers to enable the rapid self‐assembly and reflection of light across the visible and in the near‐IR spectrum. It was demonstrated that the rapid self‐assembly of this dendronized BCP was induced during filament extrusion at 200 °C within several minutes. The printed 3D cuboids, cylinders, or pyramids could reflect violet, green or red light when using block copolymers with different molecular weights. It is noteworthy that a hollow U‐shaped tube could be easily printed, which enables transformation of the white light into green light when exiting the other opening. Colloidal semiconductor nanowires have also been printed with a polystyrene–polyisoprene–polystyrene block copolymer matrix.^[^ ^260^ ^]^ The 3D printing process enables good control over the alignment of the nanowire. SAXS result suggests that the printed block copolymer filaments have a long‐range order and an anisotropic structure (**Figure** **16**A). When printing block copolymer with perovskite crystals, perovskite nanowires are oriented in parallel with the print direction and locally conform to the block copolymer microdomains as demonstrated by TEM result (Figure 16B). By combining 3D printing architectural design and highly polarized, angular dependent emission properties of the nanowires, novel optical memory devices (Figure 16C) as well as polarizer tunable multiplexed color displays (Figure 16D) were easily fabricated.

## 4 Summary and Perspective

The technology of 3D extrusion printing is rapidly growing due to the ease of use and the large variety of applications. New printing technologies, new structural designs and new materials have been a major focus of study in the field of 3D printing. The polymeric inks are the most crucial component for 3D printing, as they strongly affect the development of new printing technology and the properties of the final 3D‐printed products. Many polymer materials with a wide range of tunable and fascinating properties have emerged. However, the field is still very young and the polymeric materials that have been developed for 3D printing are still very limited. The field will be greatly enhanced by the constant and innovative development of functional polymer inks with improved printability and material properties. In this review, we have briefly described recent advances in 3D extrusion printing of polymers with advanced material properties. In the first part, the material design criteria and strategies developed for achieving or enhancing the printability of diverse polymers has been systemically reviewed. The incorporation of nanofillers represents one of the most commonly used strategies to enhance the 3D printability of a diverse range of polymers. Polymers constructed through supramolecular interactions are very suitable for 3D extrusion printing, due to the weak physical bonds that can be easily broken under applied pressure, while immediate recovery to the solid state is possible when the shear force is removed. However, in order to maintain structural stability, post‐printing treatment is usually required for enhancing the molecular interactions and mechanical properties. The printing of covalently crosslinked polymers has also been achieved, through careful tuning of the ink formulations. Despite the impressive progress that has been made to print diverse polymers using 3D extrusion methods, there are still many challenges in printing polymers. The use of nanofillers could lead to several undesired effects on viscosity, transparency, and especially the mechanical properties of the printing materials, due to the possibility of phase separation. The use of dynamic bonds which undergo reversible breaking, exchange and reformation could be utilized to modify the printability of polymers as well as imparting various advanced functions.^[^ ^41^, ^42^, ^43^, ^44^, ^45^, ^46^, ^47^, ^48^, ^49^, ^50^, ^51^, ^52^, ^53^, ^54^, ^55^, ^56^, ^57^, ^58^, ^59^, ^60^, ^61^, ^62^, ^63^, ^64^, ^65^, ^66^, ^67^, ^68^, ^69^, ^70^, ^71^, ^72^, ^73^, ^74^, ^75^, ^76^, ^77^, ^78^, ^79^, ^80^, ^81^, ^82^, ^83^, ^84^, ^85^, ^86^, ^87^, ^88^, ^89^, ^90^, ^91^, ^92^, ^93^, ^94^, ^95^, ^96^, ^97^, ^98^, ^99^, ^100^, ^101^, ^102^, ^103^, ^104^, ^105^, ^106^, ^107^, ^108^, ^109^, ^110^, ^111^, ^112^, ^113^, ^114^, ^115^, ^116^, ^117^, ^118^, ^119^, ^120^, ^121^, ^122^, ^123^, ^124^, ^125^, ^126^, ^127^, ^128^, ^129^, ^130^, ^131^, ^132^, ^133^, ^244^, ^258^, ^134^, ^135^, ^136^, ^137^, ^138^, ^139^, ^140^, ^141^, ^142^, ^143^, ^144^, ^145^, ^146^, ^147^, ^148^, ^149^, ^150^, ^151^, ^152^, ^153^, ^154^, ^155^, ^156^, ^157^, ^158^, ^159^, ^160^, ^161^, ^162^, ^163^, ^164^, ^165^, ^166^, ^167^, ^168^, ^169^, ^170^, ^171^, ^172^, ^173^, ^174^, ^175^, ^176^, ^177^, ^178^, ^179^, ^180^, ^181^, ^182^, ^183^, ^184^, ^185^, ^186^, ^187^, ^188^, ^189^, ^190^, ^191^, ^192^, ^193^, ^194^, ^195^, ^196^, ^197^, ^198^, ^199^, ^200^, ^201^, ^202^, ^203^, ^204^, ^205^, ^206^, ^207^, ^208^, ^209^, ^210^, ^211^, ^212^, ^213^, ^214^, ^215^, ^216^, ^217^, ^218^, ^219^, ^220^, ^221^, ^222^, ^223^, ^224^, ^225^, ^226^, ^227^, ^228^, ^229^, ^230^, ^231^, ^232^, ^233^, ^234^, ^235^, ^236^, ^237^, ^238^, ^239^, ^240^, ^241^, ^242^, ^243^, ^245^, ^246^, ^247^, ^248^, ^249^, ^250^, ^251^, ^252^, ^253^, ^254^, ^255^, ^256^, ^257^, ^259^, ^260^, ^261^, ^262^, ^263^ ^]^ Recent developments in polymer synthetic strategies, especially in controlled polymerization protocols^[^ ^264^, ^265^ ^]^ and novel polymer functionalization methodologies,^[^ ^266^ ^]^ could allow one to freely design advanced functional materials with almost arbitrarily controllable structures and properties. Utilizing these advanced synthetic polymer approaches may have the potential to greatly expand the polymers with excellent 3D printability. It is very challenging to use the current existing 3D extrusion printing technology to print high resolution features and hierarchical micro–nano structures. Integrating the 3D printing with the self‐assembly of polymers^[^ ^249^ ^]^ could be an efficient approach to fabricate hierarchical structures^[^ ^97^ ^]^ across different length scales, which could be very promising for numerous applications.

We have also summarized the advanced properties of polymeric objects fabricated by 3D printing. We envision significant growth in the development of multifunctional polymers for 3D printing in the future. Printed objects with integrated properties such as self‐healing, high strength, shape changing, and biocompatibility are some of the most promising candidates for 3D printing. However, most of the polymers with diverse properties suffer from structural complexity and synthetic difficulty. Therefore, preparing printable polymeric materials with integrated sophisticated properties but through inexpensive and simple routes will be an active research area. The dynamic shape changing properties of 3D printed objects achieved through excellent design of 3D printing is also discussed. For example, the soft actuators fabricated through 3D printing exhibit fast, complex, tunable, and programmable shape morphing processes, which are crucial in expanding their applications. Importantly, 3D printing technology also facilitates the precise control of the distribution of the actuation components and thus can achieve complicated 3D structures that are not accessible with traditional fabrication processes. We envision that the rapid advancement in manufacturing intricate biomimicking materials,^[^ ^5^, ^6^ ^]^ as well as the new manufacturing approaches,^[^ ^7^ ^]^ could further enable the fabrication of advanced materials with sophisticated structures and exciting properties.

We believe that there are number of opportunities in the combination of computational design of complex morphologies to create shape changing objects. With the computer‐based design of objects, practically any morphology is obtainable. However, there are many questions on what morphologies should be printed (i.e., shapes, stiffness gradients/combinations etc.). Computational modeling could lead this design to create efficient shape changing objects.

Finally, we would like to emphasize that the environmental impact should be considered. With 3D printing there is the potential to further increase society's usage of plastics. As polymer chemists, it is up to us to design sustainable and renewable materials that will not exacerbate the already significant issues of plastics in the environment. Some current materials are like polylactide are biodegradable. However, we should aspire to engineer materials that are not just biodegradable but are also renewably sourced and recyclable.

## Conflict of Interest

The authors declare no conflict of interest.
